# Comparative effectiveness of electroencephalogram-neurofeedback training of 3–45 frequency band on memory in healthy population: a network meta-analysis with systematic literature search

**DOI:** 10.1186/s12984-025-01634-8

**Published:** 2025-04-24

**Authors:** Wen-Hsiu Yeh, Ya-Ju Ju, Fu-Zen Shaw, Yu-Ting Liu

**Affiliations:** 1 Department of Gerontological Health Care, Central Taiwan University of Science and Technology, Taichung City, 406 Taiwan; 2 Teaching and Research Center, Kaohsiung Municipal Siaogang Hospital, Kaohsiung Medical University Hospital, Kaohsiung Medical University, Kaohsiung, 812 Taiwan; 3 Department of Physical Therapy, College of Health Care, China Medical University, Taichung, 406 Taiwan; 4 Department of Psychology, National Cheng Kung University, Tainan, 701 Taiwan; 5 Mind Research and Imaging Center, National Cheng Kung University, Tainan, 701 Taiwan; 6 Department of Medical Science Industries, Chang Jung Christian University, Tainan, 711 Taiwan; 7 Bachelor Degree Program in Medical Sociology and Health Care, Chang Jung Christian University, Tainan, 711 Taiwan

**Keywords:** Biofeedback, Cognition, Electroencephalographic, Memory, Neurofeedback, Network meta-analysis

## Abstract

**Objective:**

To investigate which brain activity frequency of electroencephalogram (EEG)-neurofeedback training (NFT) was the most effective for enhancing working memory (WM) and episodic memory (EM) in healthy participants through network meta-analysis (NMA).

**Methods:**

Searched PubMed, Embase, and Cochrane Library for studies published from January 1990 to January 2025. We performed Bayesian NMA, pooling continuous outcome data using the standardized mean difference effect size (ES). Global and local evaluations of inconsistency were conducted using the chi-square test, side-splitting, and loop-specific approaches. A consistency model was applied and the global approach to inconsistency showed no significance. Efficacy ranks were determined using the surface under the cumulative ranking curve (SUCRA) for each intervention. Publication bias was assessed using the comparison-adjusted funnel plot and Egger’s test. Finally, sensitivity analysis confirmed our findings’ robustness.

**Results:**

Sixty studies were included, comprising 50 trials on WM and 24 trials on EM. While the global inconsistency analysis showed no significant inconsistency for WM (χ^2^(22) = 30.89, *p* = 0.10) and EM (χ^2^(10) = 13.48, *p* = 0.19), the consistency model exhibited the most significant difference between active control (AC) and alpha combined with working memory training (WMT) (ES of 6.64, *p* < 0.001) for WM, and between AC and alpha (ES of 0.84, *p* = 0.01) for EM. Alpha combined with WMT for WM (100%) and alpha NFT for EM (87.0%) also showed the highest efficacy according to the SUCRA. No publication bias was found for either type of memory. The sensitivity analysis for WM and EM aligns with the original results.

**Conclusion:**

Through NMA, alpha activity (7–13 Hz) may be a crucial frequency impacting memory. Brain activity combined with other training methods requires more robust studies for future investigation. This study registered with www.crd.york.ac.uk/prospero/ (CRD42024539656).

**Supplementary Information:**

The online version contains supplementary material available at 10.1186/s12984-025-01634-8.

## Introduction

Electroencephalogram (EEG)-Neurofeedback training (NFT) represents a relatively new advancement in the field of neuropsychological rehabilitation for brain training, progressively advocated since the 1990s [1]. Through a biofeedback model, EEG-NFT enables participants to learn how to enhance the electrical activity of specific brain frequencies. Participants wear a recording apparatus that captures and displays their brain activities as a feedback signal. This feedback allows participants to visualize their brain activities, self-regulate their brain responses, and develop effective strategies to increase specific brain activities. To date, various EEG-NFT protocols have been employed across studies, targeting different frequency bands: theta (3–8 Hz), alpha (7–13 Hz), including upper alpha (UA) (10–13.5 Hz), beta (11.6–20 Hz), including sensorimotor rhythm (SMR) (12–15 Hz) and low beta (LB) (15–18 Hz), and gamma (30–45 Hz). Each frequency band affects different brain areas, i.e., theta primarily influencing the frontal sites, alpha the parietal-occipital sites, beta the central sites, and gamma the parieto-occipital sites [2]. These frequencies oscillations are related to govern cognitive processes [3] and highlight the functional roles of each frequency band in memory [4–6]. To determine which frequency band is the most effective in EEG-NFT for enhancing memory and to reduce substantial heterogeneity in the selected population, a healthy population was well selected for this study.

EEG-NFT targeting theta, alpha, beta, and gamma activities has demonstrated significantly positive effects on working memory (WM) and episodic memory (EM) in healthy participants [4–7]. Recent studies also show that EEG-NFT is effective for improving memory in elder [8] and clinical subjects [2]. However, control groups in EEG-NFT studies have not been clearly delineated, with variations including silent control (SC), sham, active control (AC), or passive control (PC) groups, leading to inconsistent results across studies [9–12]. For instance, comparisons between SC and EEG-NFT on memory indicate significant differences [10, 13], whereas comparisons between sham and EEG-NFT show no significant influence on memory [14, 15]. Additionally, some studies demonstrate well-designed experiments between AC and NFT groups [16, 17], but others report no significant differences between these groups [18, 19]. Overall, while EEG-NFT has shown positive effects on memory across different frequency bands, the primary frequency band affecting the intensity of WM and EM remains unexplored. Furthermore, a well-controlled group is essential to clarify the potential efficacy of EEG-NFT on memory.

To address the aforementioned issues, we systematically screened all previous studies related to NFT of 3–45 frequency activity on memory in healthy participants, following the Preferred Reporting Items for Systematic Reviews and Meta-Analysis (PRISMA) guidelines for literature search and selection. We summarized and classified the characteristics of each study based on the category of trained brain activity (i.e., theta, alpha, UA, beta, SMR, LB, and gamma) and control protocols (i.e., SC, sham, AC, and PC). Subsequently, we conducted a network meta-analysis (NMA) using both new techniques and existing studies to investigate the effects of EEG-based NFT on WM, which is defined as memory that is active and relevant only for a short period of time [20], and EM, which is a longer-lasting memory that allows one to recall and re-experience personal events. Both types of memory are involved in the interaction between performing an ongoing task (e.g., a day’s work) and the flexible encoding, retrieval, and realization of a prospective task [21]. Moreover, EM contributes to WM bindings [22]. Hence, we focus on investigating WM and EM. Additionally, this study provides a comprehensive quantitative overview, assessing which frequency band of brain activity is the most viable training approach for WM and EM in healthy populations and identifying the most effective control approach for EEG-based NFT studies.

## Materials and methods

This study adhered to the PRISMA guidelines for conducting the search and analysis of international scientific literature [23]. The study was registered in the International Prospective Register of Systematic Reviews (PROSPERO) with the registration number CRD42024539656.

### Data sources and searches

Three electronic bibliographic databases—PubMed, Embase, and The Cochrane Library—were utilized for the literature search, covering the period from January 1, 1990, to January 31, 2025. To ensure a wide range of EEG-NFT studies, the PICOS framework was employed. The search string focused on intervention (I = neurofeedback), and outcome (O = memory and cognition), while population (P), comparison (C) and study design (S) were not specified. The search terms used were: (neurofeedback OR Neurofeedback OR Electroencephalographic biofeedback OR electroencephalographic biofeedback OR EEG biofeedback OR EEG Biofeedback) AND (memor* OR Memor* OR cogniti* OR Cogniti*).

### Study selection

The reference manager software Clarivate EndNoteTM 20 was utilized to combine search results and delete duplicate records. Two investigators (W.-H.Y. and Y.-J.J.) independently screened the literature from the three databases to identify potentially relevant studies according to the inclusion and exclusion criteria. Any discrepancy was solved through discussion with the presence of a third author (F.-Z.S.) to reach a consensus on every relevant detail.

The inclusion criteria were as follows:

1. Research design involving a randomized controlled trial or comparative study.
2. Healthy participants with no any neurological disorder, psychological disorders, and objective evidence of memory dysfunction.
3. The control group received: (a) SC involved only maintaining activities of daily living without receiving any intervention, or just receiving pre- and post-assessments; (b) sham neurofeedback involved a fake or inert EEG activity stimulation, either from other participants or replaying a brain activity video from previous training, without actual brainwave modulation during the training; (c) AC involved a randomly selected non-trained amplitude from a range of the participant’s brain activity (e.g., 2 Hz randomly from the participant’s 2–12 Hz [24], 2 Hz from their 10–24 Hz [11, 25], 2 Hz from their 1–30 Hz [26], 3 Hz from their 16–24 Hz [27], 4 Hz from their 7–20 Hz [16, 17, 28], 4 Hz from their 4–45 Hz [29], or 4–6 Hz randomly from their 10–25 Hz [30]), down-regulation of non-trained brain activity [31], trained brain activity from up to down regulation [19], or non-responder brain activity [18, 32], which differs from sham by providing real-time feedback based on the active self-modulation of participant’s brain activity, and without any targeted modulation or training of specific brain regions; d.) PC involved just looking at something (e.g., watching a movie [33], target search [34], playing a Sudoku game [35], or viewing a graphical representation [36]), listening to something (i.e., pitch [12]), or audio-visual combined stimulation [37].
4. The intervention was EEG-based brain activity NFT involving theta (3–8 Hz), alpha (7–13 Hz) including UA (10–13.5 Hz), beta (12–22 Hz) including SMR (11.6–16 Hz) and LB (15–18 Hz), gamma (30–45 Hz), training of both brain-activity ratios, or brain activity combined with another brain activity, PC, or working memory training (WMT).
5. The evaluation assessed the effects of EEG-based NFT on WM involving the temporary storage and manipulation of information [38] (i.e., backward/forward digit span tests (B/FDSTs), Corsi block test (CBT), culture fair intelligence test (CFIT), delayed matching to sample task (DMTST), eight-word repetition task-immediate recall (EWRT-ir), Kaufman brief intelligence test 2 & wide range intelligence test (K-BIT 2 & WRIT), Kim Karad visual memory test-short term (KKVMT-st), mental rotation test (MRT), modified Sternberg test (MST), N-back task, object decision test (ODT), Rey auditory verbal learning test-immediate recall (RAVLT-ir), semantic working memory test (SWMT), trail making test-B (TMT-B), visuomotor binding cost (VMBS), verbal memory test (VMT), visuo-spatial memory test (VSMT), Wechsler adult intelligence scale-III (WAIS-III), 2 or 3 back tasks, 3-dimensional multiple object tracking (3D-MOT)), and/or EM involving the ability to encode, consolidate, and retrieve past events along with their contextual details [39] (i.e., cognitive change index-self report (CCI-S), Graphic pair test (GPT), movie plot-delayed recall (MP-dr), NEUROPSI-memory, Rey auditory verbal learning test-delayed recall (RAVLT-dr), visual memory-delayed recall (VM-dr), visual and verbal memory test 2-delayed recall (VVMT2-dr), Wechsler memory scale-R (WMS-R), word pair test (WPT)).

The exclusion criteria were followed if the articles were non-full-text articles (i.e., abstracts, conference abstracts, e-posters, posters, letters, author’s reply, forum, opinion, or registered clinical trials), not research articles (e.g., review, book, opinion, or letter), or not written in English. If data or full-text availability was lacking, the corresponding author was contacted via email; if no reply was received, these studies were excluded from the NMA. To prevent the natural processes from affecting the intervention [40], studies with data of change (post-pre training) or without post-training data were excluded.

### Data extraction and statistical analysis

One author (W.-H.Y.) extracted data from the selected trials, and another author (Y.-J.J.) checked the accuracy. Subsequently, W.-H.Y. verified the numerical results for the accuracy of statistical information. Data were recorded using a standardized data extraction form, which included the following properties of each study in a database: study characteristics (sample size, category of EEG-based brain-activity NFT, number of participants) and participant details (age). EEG-NFT effects on WM and EM were collected as outcomes of interest.

Mean and standard deviation (SD) values were used to calculate the effect size. Data were extracted from the groups at the post-evaluation. If the article reported data as standard error (SE), the formula ($\:\text{S}\text{E}=\text{S}\text{D}/\surd\:\text{n}\text{u}\text{m}\text{b}\text{e}\text{r}\:\text{o}\text{f}\:\text{s}\text{a}\text{m}\text{p}\text{l}\text{e}\text{s}$) was used to convert SE into SD. Additionally, a higher score represented better memory performance in the extracted data. If the data exhibited a lower score representing good performance, the following formula ($\:(SD/mean$)*100) was used for standardization in some studies [24, 41]. Some studies reported error rates [42–44], a calculation of 100-error rate was used.

The standardized mean difference (SMD) for continuous outcomes with 95% confidence intervals (CIs) was calculated for NMA. An overview of the available evidence and the network map of eligible comparisons of EEG-based brain activity NFT on WM and EM was performed. The nodes were weighted based on the number of studies involving the respective interventions, while the lines were weighted based on the number of participants according to the evaluation of the intervening comparisons. To evaluate inconsistency within the network, the global approach was assessed a priori using the chi-square (χ^2^) test. Then, local inconsistency between direct and indirect evidence was evaluated using node-splitting models and the loop-specific approach. A 95% confidence interval (CI) in a loop that includes zero suggests that there is no significant inconsistency. Additionally, a consistency model based on the comparative efficacy in SMD effect size with CI was evaluated. If the inconsistency in the global/local approaches was tested and found to be non-significant, the intervention efficacy was accepted. The surface under the cumulative ranking curves (SUCRAs) was calculated to rank treatments for each independent outcome. The SUCRA values range from 0 to 100%. A higher SUCRA value indicates a more effective intervention, whereas a value closer to zero indicates an intervention of bottom rank. Publication bias was examined using a comparison-adjusted funnel plot with Egger’s test for detection. A sensitivity analysis was performed to evaluate the robustness of our findings by excluding the study with the smallest sample size for WM and EM, respectively. The Stata software package (version 17, StataCorp, College Station, Texas, United States) was used for all statistical analyses, with the significance level set at *p*-value < 0.05, except for when comparing direct and indirect evidence in a z-test, where the significance level was set at *p*-value < 0.10 [45].

## Results

### Study selection and characteristics

**Fig. 1:**
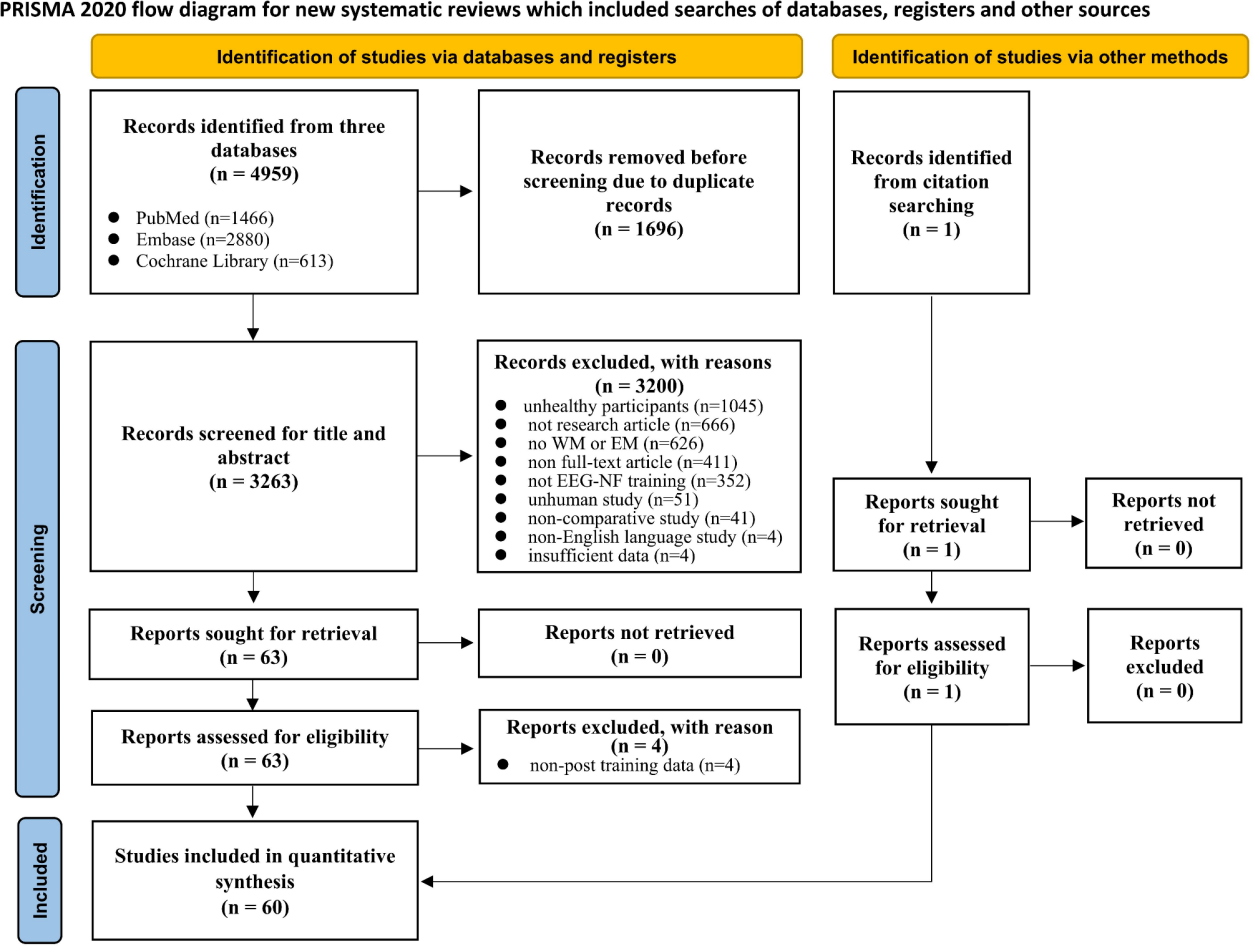
The PRISMA flowchart outlining the selection of studies included

Figure 1 shows the PRISMA 2020 schematic flow diagram for the process of study selection. A total of 4959 titles and abstracts were initially identified, with 1696 removed due to duplicates, and 3263 screened for title and abstract. Of these, 3200 were excluded for not following the inclusion criteria. Sixty-three studies were sought for retrieval and assessed for eligibility from the databases. However, 4 studies were excluded due to the absence of post-training data. Finally, 59 studies, along with an additional study identified through citation searching, were included, making a total of 60 studies for the quantitative synthesis and analyses.

Table 1 shows the characteristics of each study. The studies with EEG-based brain activity NFT on memory were published between 2003 and 2024. The studies included healthy participants, with an accumulated sample size of 1995, ranging from 8 to 120. The mean age of the overall population was ~ 30.84±16.61 years. Additionally, 50 of the 60 studies (83.3%) included WM assessment: BDST and MRT were used in 9 studies each, 3-back task in 7 studies, n-back task in 3 studies, DMTST, FDST, MST and 2-back task in 2 studies each, and CBT, CFIT, EWRT-ir, K-BIT 2 & WRIT, KKVMT-st, ODT, RAVLT-ir, SWMT, TMT-B, VMBS, VMT, VSMT, WAIS-III, 3D-MOT in 1 study each. For EM, 24 of the 60 studies (40%) included assessment: WPT was used in 7 studies, GPT in 6 studies, RAVLT-dr and VVMT2-dr in 3 studies each, and CCI-S, MP-dr, NEUROPSI-memory, VM-dr, WMS-R in 1 study each.

**Table 1:** Characteristics of studies included in network Meta-Analysis

References	Characteristic of EEG-NFT	OOIs	
(Author, years)	Group (*n*, mean±SD)	WM: mean±SD	EM: mean±SD
**Alekseeva, 2012** [84]	**Total** ***n*** = **27** Sham (*n* = 13, 19.8 ± 0.6) UA (*n* = 14, 19.5 ± 0.4)	**MRT**: 39 ± 7.5 48 ± 7.5	
**Andrade**,** 2024** [78]	**Total** ***n*** = **31** Sham (*n* = 14, 73.0 ± 5.5) Gamma (*n* = 17, 71.0 ± 7.6)	**BDST**: 4.93 ± 5.16 5.82 ± 3.92	
**Becerra**,** 2012** [9]	**Total** ***n*** = **14** Sham (*n* = 7, 67 ± 4.9) Theta (*n* = 7, 65.8 ± 2.4)	**WAIS-III**: 96 ± 11 107 ± 6	**NEUROPSI-memory**: 107 ± 11 110 ± 12
**Berner**,** 2006** [14]	**Total** ***n*** = **11**,** 20.8 ± 2.8 (crossover)** Sham (*n* = 11) SMR (*n* = 11)		**WPT**: 40.97 ± 22.31 41.82 ± 19.73
**Brandmeyer**,** 2020** [41]	**Total** ***n*** = **24** Sham (*n* = 12, 25 ± 3) Theta (*n* = 12, 25 ± 3)	**N-back task**: 1.07 ± 0.03 1.07 ± 0.03	
**Campos da Paz**,** 2018** [10]	**Total** ***n*** = **17**,** 69.05 ± 2.1** SC (*n* = 4) Sham (*n* = 6) SMR (*n* = 7)	**DMTST**: 8.5 ± 2.51 16.5 ± 5.35 20 ± 2.93	
**Chen**,** 2023** [74]	**Total** ***n*** = **16**,** 24.28 ± 1.31** SC (*n* = 8) Alpha (*n* = 8)	**3-back task**: 70.75 ± 4.62 75.69 ± 3.94	
**Chikhi**,** 2023** [37]	**Total** ***n*** = **85** PC (*n* = 39, 23.28 ± 5.96) UA (*n* = 46, 22.33 ± 5.62)	**3-back task**: 80.27 ± 9.66 80.53 ± 9.69	
**Chikhi**,** 2024** [26]	**Total** ***n*** = **101** AC (*n* = 34, 21.41 ± 8.28) Theta (*n* = 28, 19.40 ± 1.81) UA (*n* = 39, 23.28 ± 5.96)	**3-back task**: 82 ± 8 80 ± 9 81 ± 7	
**Diotaiuti**,** 2024** [36]	**Total** ***n*** = **40**,** aged 19–26 years** PC (*n* = 20) Alpha (*n* = 20)_	**CBT**: 5.7 ± 0.83 5.65 ± 1.70	
**Domingos**,** 2020** [79]	**Total** ***n*** = **30** SC (*n* = 15, 22.53 ± 3.89) Alpha (*n* = 15, 27.93 ± 6.11)	**FDST**: 7.93 ± 0.96 8.13 ± 0.83	
**Domingos**,** 2021a** [75]	**Total** ***n*** = **30** SC (*n* = 15, 21.20 ± 2.62) Alpha (*n* = 15, 22.53 ± 3.89)	**N-back task**: 96.00 ± 6.32 98.67 ± 8.84	
**Domingos**,** 2021b** [77]	**Total** ***n*** = **30**,** aged 18–34 years** SC (*n* = 15) Alpha (*n* = 15)	**N-back task**: 96.00 ± 6.31 98.33 ± 3.09	
**Enriquez-Geppert**,** 2014** [67]	**Total** ***n*** = **40** Sham (*n* = 21, 25.8 ± 3.8) Theta (*n* = 19, 23.8 ± 2.7)	**3-back task**: 80 ± 4.6 89 ± 4.4	
**Eschmann**,** 2020** [11]	**Total** ***n*** = **35** AC (*n* = 18, 23.30) Theta (*n* = 17, 22.65)		**WPT**: 59.0 ± 12.72 64.5 ± 20.61
**Eschmann**,** 2021** [25]	**Total** ***n*** = **35** AC (*n* = 18, 23.30) Theta (*n* = 17, 22.65)	**DMTST**: 97.9 ± 2.12 98.9 ± 2.06	
**Escolano**,** 2012** [85]	**Total** ***n*** = **19** Sham (*n* = 9, 24.3 ± 3.67) UA (*n* = 10, 25.8 ± 4.07)	**MRT**: 82 ± 6 90 ± 4.74	**RAVLT-dr**: 14.5 ± 0.7 14.8 ± 0.6
**Escolano**,** 2014** [86]	**Total** ***n*** = **19** Sham (*n* = 9, 24.3 ± 3.7) UA (*n* = 10, 28.5 ± 4.1)	**MRT**: 41.30 ± 1.95 45.20 ± 3.79	**RAVLT-dr**: 14.56 ± 1.62 14.8 ± 1.11
**Farnia**,** 2017** [87]	**Total** ***n*** = **30** Sham (*n* = 10, 34.2 ± 5.70) LA/UA (*n* = 10, 31.7 ± 6.40) Beta (*n* = 10, 31.70 ± 6.65)		**WMS-R**: 107.26 ± 7.05 117.68 ± 7.04 122.46 ± 7.02
**Farraj**,** 2024** [73]	**Total** ***n*** = **57** SC (*n* = 32, 23.8 ± 2.37) UA (*n* = 25, 25.1 ± 2.21)	**MRT**: 91.60 ± 8.49 90.30 ± 9.70	
**Fritson**,** 2007** [15]	**Total** ***n*** = **32**,** 21.3 ± 4.24** Sham (*n* = 16) SMR (*n* = 16)	**K-BIT 2 & WRIT**: 106.13 ± 10.8 106.00 ± 12.5	
**Gordon**,** 2019** [34]	**Total** ***n*** = **120** SC (*n* = 40, 21.98 ± 2.51) UA (*n* = 20, 21.65 ± 2.45) UA + WMT (*n* = 20, 21.60 ± 1.93) UA + PC (*n* = 20, 22.60 ± 2.93) PC (*n* = 20, 21.47 ± 2.35)	**MRT**: 75.00 ± 13.00 72.00 ± 15.00 83.00 ± 11.00 71.00 ± 15.00 75.00 ± 12.00	
**Guez**,** 2014** [65]	**Total** ***n*** = **30**,** 23.63 ± 2.79** Sham (*n* = 10) UA (*n* = 10) SMR (*n* = 10)	**VMT**: 97.00 ± 4.83 96.77 ± 8.21 98.22 ± 2.04	**WPT**: 99.00 ± 3.16 96.66 ± 5.00 97.77 ± 6.66
**Hanslmayr**,** 2005** [18]	**Total** ***n*** = **18**,** 24.85 ± 3.55** AC (*n* = 9) UA (*n* = 9)	**MRT**: 57.5 ± 15 62.5 ± 13.5	
**Hsueh**,** 2016** [16]	**Total** ***n*** = **50** AC (*n* = 25, 21.64 ± 2.40) Alpha (*n* = 25, 20.96 ± 2.85)	**BDST**: 93.24 ± 4.84 96.10 ± 3.56	**WPT**: 68.05 ± 13.90 85.06 ± 9.51
**Hsueh**,** 2012** [28]	**Total** ***n*** = **70** AC (*n* = 22, 20.6 ± 2.8) Alpha (*n* = 25, 21.6 ± 2.4) SMR (*n* = 23, 21.3 ± 2.3)	**BDST**: 95 ± 5.63 96 ± 2.5 93.5 ± 9.6	**WPT**: 70 ± 14.1 85 ± 5 80 ± 9.6
**Keizer**,** 2010a** [88]	**Total** ***n*** = **17**,** 22.6 years** Beta (*n* = 9) Gamma (*n* = 8)		**GPT**: 75.5 ± 9 80.1 ± 11.3
**Keizer**,** 2010b** [24]	**Total** ***n*** = **14**,** 22 years** AC (*n* = 7) Gamma (*n* = 7)	**VMBC**: 40.69 ± 10.58 119.1 ± 11.91	
**Khodakarami**,** 2020** [31]	**Total** ***n*** = **8** AC (*n* = 2, 25.69 ± 0.74) Gamma (*n* = 6, 24.05 ± 2.61)	**CFIT**: 33.00 ± 7.07 51.33 ± 8.64	
**Kober**,** 2015** [66]	**Total** ***n*** = **20**,** 24.40 ± 8.27** Sham (*n* = 10) SMR (*n* = 10)	**BDST**: 9.80 ± 1.89 8.80 ± 1.49	**VVMT2-dr**: 49.40 ± 7.94 48.70 ± 8.66
**Kober**,** 2017** [80]	**Total** ***n*** = **20** SMR (*n* = 10, 46.80 ± 6.29) Gamma (*n* = 10, 46.00 ± 3.98)	**BDST**: 9.50 ± 1.08 9.50 ± 2.06	**VVMT2-dr**: 56.10 ± 9.29 46.00 ± 10.25
**Kober**,** 2020** [19]	**Total** ***n*** = **20** AC (*n* = 10, 25.30 ± 2.16) SMR (*n* = 10, 24.5 ± 2.22)	**BDST**: 9.00 ± 1.93 8.30 ± 1.71	**VVMT2-dr**: 48.50 ± 7.08 44.70 ± 11.86
**Lecomte**,** 2011** [89]	**Total** ***n*** = **20**,** 75.25 years** SC (*n* = 10) UA (*n* = 10)	**EWRT-ir**: 11.10 ± 1.10 11.50 ± 0.71	**GPT**: 10.40 ± 1.17 11.10 ± 0.99
**Li**,** 2023** [90]	**Total** ***n*** = **32** Sham (*n* = 16, 21.19 ± 2.56) Alpha (*n* = 16, 22.12 ± 3.31)	**MRT**: 78.69 ± 13.58 77.00 ± 22.37	
**Marcos-Martínez**,** 2023** [27]	**Total** ***n*** = **19**,** 25.05 ± 4.18** AC (*n* = 8) Theta (*n* = 11)	**3-back task**: 76 ± 26.87 77 ± 16.58	
**Naas**,** 2019** [68]	**Total** ***n*** = **33**,** 21.27 ± 1.43** Sham (*n* = 16) UA (*n* = 17)	**FDST**: 9.69 ± 1.96 10.06 ± 2.49	
**Nan**,** 2012** [52]	**Total** ***n*** = **32**,** 23.28 ± 3.11** SC (*n* = 16) Alpha (*n* = 16)	**BDST**: 10.25 ± 1.98 10.44 ± 3.53	
**Nawaz**,** 2023** [42]	**Total** ***n*** = **36**,** 25.45 ± 5.36** SC (*n* = 19) Alpha (*n* = 17)	**2-back task**: 87.58 ± 4 90.66 ± 8	
**Nazer**,** 2018** [91]	**Total** ***n*** = **26** SC (*n* = 14, 22.28 ± 1.93) SMR (*n* = 12, 22.58 ± 1.56)	**KKVMT-st**: 2 ± 0.96 2.41 ± 0.66	**VM-dr**: 2.1 ± 0.61 2.1 ± 0.39
**Paban**,** 2024** [92]	**Total** ***n*** = **25** AC(*n* = 12, 70.50 ± 7.62) Alpha (*n* = 13, 72.46 ± 6.25)		**CCI-S**: 27.26 ± 8.12 26.15 ± 4.33
**Parsons**,** 2021** [93]	**Total** ***n*** = **30** SC (*n* = 10, 23.02 ± 2.78) Sham + WMT (*n* = 10, 23.10 ± 3.48) Alpha + WMT (*n* = 10, 21.90 ± 2.99)	**3D-MOT**: 43.5 ± 4.5 55 ± 5 72 ± 3	
**Pei**,** 2018** [29]	**Total** ***n*** = **20** AC (*n* = 10, 21.2 ± 1.72) Alpha (*n* = 10, 22.7 ± 1.95)	**BDST**: 73.28 ± 8.39 82.83 ± 10.87	**WPT**: 54.00 ± 20.73 53.13 ± 27.05
**Rozengurt**,** 2023** [35]	**Total** ***n*** = **60**,** 31.8 ± 7.60** PC (*n* = 20) Theta/LB (*n* = 20) Beta/theta (*n* = 20)		**MP-dr** 81.5 ± 13.42 78.5 ± 6.71 81.5 ± 13.42
**Rozengurt**,** 2017** [33]	**Total** ***n*** = **75** PC (*n* = 25, 28.8 ± 6.5) Theta (*n* = 25, 30.9 ± 7.7) Beta (*n* = 25, 28.6 ± 7.0)		**GPT**: 18.2 ± 6 28 ± 1 23 ± 4
**Salari**,** 2012** [32]	**Total** ***n*** = **20**,** 32 years** AC (*n* = 10) Gamma (*n* = 10)		**GPT**: 59.70 ± 13.38 66.76 ± 12.46
**Salari**,** 2014** [94]	**Total** ***n*** = **24**,** 29 years** Sham (*n* = 12) Gamma (*n* = 12)	**ODT**: 72.5 ± 15.59 87 ± 9.01	
**Shen**,** 2023** [76]	**Total** ***n*** = **25**,** 22.90 ± 1.47** SC (*n* = 10) Alpha (*n* = 15)	**3-back task**: 44.7 ± 2.11 45.5 ± 1.64	
**Shtoots**,** 2021** [95]	**Total** ***n*** = **54** SC (*n* = 18, 24.8 ± 3.7) Theta (*n* = 18, 22.8 ± 2.9) LB (*n* = 18, 24.1 ± 3.4)	**VSMT**: 65.98 ± 14.31 66.19 ± 22.46 67.13 ± 12.69	
**Smit**,** 2023** [69]	**Total** ***n*** = **55** Sham (*n* = 28, 32.5 ± 9.8) Theta (*n* = 27, 34.5 ± 11.8)	**3-back task**: 81 ± 10.58 79 ± 10.39	
**Staufenbiel**,** 2014** [96]	**Total** ***n*** = **20** Beta (*n* = 10, 66.4 ± 6.01) Gamma (*n* = 10, 69.2 ± 5.91)		**GPT**: 81.1 ± 10.75 78.4 ± 10.75
**Tseng**,** 2021** [12]	**Total** ***n*** = **32**,** 21.60 ± 4.15** PC (*n* = 15) Theta/LB (*n* = 17)		**GPT**: 62 ± 11.62 68 ± 20.62
**Uslu**,** 2023** [43]	**Total** ***n*** = **29**,** 24.65 years** Sham (*n* = 10) UA (*n* = 19)	**MRT**: 90.43 ± 4.96 87.86 ± 8.76	
**Van Eijk**,** 2017** [71]	**Total** ***n*** = **16** SC (*n* = 6, 79.2 ± 6.9) SMR (*n* = 10, 77.9 ± 7.8)	**RAVLT-ir**: 34.7 ± 13.4 41.6 ± 15.7	**RAVLT-dr**: 6.0 ± 4.2 9.5 ± 4.8
**Vekety**,** 2022** [97]	**Total** ***n*** = **28**,** 9.92 ± 4.35** SC (*n* = 15) Alpha + Theta (*n* = 13)	**TMT-B**: 61.2 ± 14.7 60.1 ± 22.6	
**Vernon**,** 2003** [98]	**Total** ***n*** = **30**,** 22.1 ± 1.77** SC (*n* = 10) Theta (*n* = 10) SMR (*n* = 10)	**SWMT**: 74.5 ± 9.49 75.5 ± 9.49 82 ± 12.65	
**Wang**,** 2013** [30]	**Total** ***n*** = **16** AC (*n* = 8, 64.6 ± 2.4) Theta (*n* = 8, 65 ± 3.3**)**	**MST**: 84 ± 28.3 91 ± 17.0	
**Wei**,** 2017** [17]	**Total** ***n*** = **30**,** 26 ± 3** AC (*n* = 15) Alpha (*n* = 15)	**BDST**: 91 ± 3.09 97 ± 5.81	**WPT**: 58 ± 15.49 81 ± 10.45
**Xiong**,** 2014** [13]	**Total** ***n*** = **36**,** young adult** SC (*n* = 12) Sham (*n* = 12) Theta/alpha (*n* = 12)	**2-back task**: 82.94 ± 7.79 81.70 ± 12.33 90.69 ± 4.83	
**Zhou**,** 2024** [44]	**Total** ***n*** = **40** Sham (*n* = 20, 23.55 ± 1.28) Alpha (*n* = 20, 24.10 ± 1.21)	**MST** 72 ± 11.18 70 ± 13.41	
**Zoefel**,** 2011** [72]	**Total** ***n*** = **22** SC (*n* = 10, 22.1 ± 3.8) UA (*n* = 12, 23.7 ± 2.3)	**MRT**: 114.7 ± 19.08 129.7 ± 11.63	

Abbreviations:

a.) related to EEG-NFT: *AC*, active control; *EEG*, electroencephalogram; *LA*, lower alpha; *LB*, lower beta; *NFT*, neurofeedback training; *PC*, passive control; *SC*, silent cotnrol; *SD*, standard deviation; *SMR*, sensorimotor rhythm; *UA*, upper alpha; *WMT*, working memory training.

b.) related to OOIs: *BDST*, backward digit span test; *CBT*, Corsi block test; *CCI-S*, cognitive change index-self report; *CFIT*, culture fair intelligence test; *DMTST*, delayed matching to sample task; *EM*, episodic memory; *EWRT-ir*, eight-word repetition task-immediate recall; *FDST*, forward digit span test; *GPT*, Graphic pair test; *K-BIT*, Kaufman brief intelligence test; *KKVMT-st*, Kim Karad visual memory test-short term; *MP-dr*, movie plot-delayed recall; *MRT*, mental rotation test; *MST*, modified Sternberg test; *ODT*, object decision test; *OOIs*, outcome of interests; *RAVLT-dr*, Rey auditory verbal learning test-delayed recall; *RAVLT-ir*, Rey auditory verbal learning test-immediate recall; *SWMT*, semantic working memory test; *TMT*, trail making test; *VM-dr*, visual memory-delayed recall; *VMBC*, visuomotor binding cost; *VMT*, verbal memory test; *VSMT*, visuo-spatial memory test; *VVMT-dr*, visual and verbal memory test-delayed recall; *WAIS*, Wechsler adult intelligence scale; *WM*, working memory; *WMS*, Wechsler memory scale; *WPT*, word pair test; *WRIT*, wide range intelligence test; *3D-MOT*, 3-dimensional multiple object tracking

Table 2 shows the classification of intervention model and definition of EEG-NFT. The control groups were related to SC including 19 articles (19/60 = 31.7%), sham including 20 articles (20/60 = 33.3%), AC including 15 articles (15/60 = 25.0%), and PC including 6 articles (6/60 = 10.0%). In regard to NFT of brain activity, 12 articles (12/60 = 20.0%) involve theta activity (3–7 Hz), 15 articles (15/60 = 25.0%) involve alpha activity (7–13 Hz), 13 articles (13/60 = 21.7%) involve UA (10–13.5 Hz), 4 articles (4/60 = 6.7%) involve beta (12–22 Hz), 11 articles (11/60 = 18.3%) involve SMR (11.6–16 Hz), 1 article (1/60 = 1.7%) involve LB (15–18 Hz), and 8 articles (8/60 = 13.3%) involve gamma (30–45 Hz). Additionally, NFT of both brain-activity ratios involves 5 articles (5/60 = 8.3%), 1 with LA (7–9.5 Hz)/UA (9.5–12 Hz), 1 with theta (4–7 Hz)/alpha (8–12 Hz), 2 with theta (4–8 Hz)/LB (14–18 Hz), and 1 with beta (15–22 Hz)/theta (4–8 Hz). The mixed training involves 5 articles (5/60 = 8.3%), 1 with sham + WMT, 1 with alpha (8–13 Hz) + WMT, 1 with alpha (8–12 Hz) + theta (4–8 Hz), 1 with UA (10–12 Hz) + WMT, as well as 1 with UA (10–12 Hz) + PC.

**Table 2:** Classification of the intervention models of EEG-NFT in the included studies

Intervention models	Definition	References
**A. Control group**		
**1. SC**	involving with only maintaining activities of daily living, not receiving any intervention, or just receiving pre and post assessments.	(Campos, 2018; Chen, 2023; Domingos, 2020; Domingos, 2021a; Domingos, 2021b; Farraj, 2024; Gordon, 2019; Lecomte, 2011; Nan, 2012; Nawaz, 2023; Nazer, 2018; Parsons, 2021; Shen, 2023; Shtoots, 2021; Van Eijk, 2017; Vekety, 2022; Vernon, 2003; Xiong, 2014; Zoefel, 2011) [10, 13, 34, 42, 52, 71–77, 79, 89, 91, 93, 95, 97, 98]
**2. Sham**	involving with a fake or inert EEG activity stimulation, either from other participants or replaying a BA video from previous training, without actual brain activity modulation during the training.	(Alekseeva, 2012; Andrade, 2024; Becerra, 2012; Berner, 2006; Brandmeyer, 2020; Campos da Paz, 2018; Enriquez-Geppert, 2014; Escolano, 2014; Escolano, 2012; Farnia, 2017; Fritson, 2007; Guez, 2014; Kober, 2015; Li, 2023; Naas, 2019; Salari, 2014; Smit, 2023; Uslu, 2023; Xiong, 2014; Zhou, 2024) [9, 10, 13–15, 41, 43, 44, 65–69, 78, 84–87, 90, 94]
**3. AC**	involving with real-time feedback based on the active self-modulation of participant’s brain activity, and without any targeted modulation or training of specific brain regions.	(Chikhi, 2024; Eschmann, 2020; Eschmann, 2021; Hanslmayr, 2005; Hsueh, 2016; Hsueh, 2012; Keizer, 2010b; Khodakarami, 2020; Kober, 2020; Marcos-Martínez, 2023; Paban, 2024; Pei, 2018; Salari, 2012; Wang, 2013; Wei, 2017) [11, 16–19, 24–32, 92]
**4. PC**	involving with just looking at the something, listening on the something, or audio-visual combined stimulation.	(Chikhi, 2023; Diotaiuti, 2024; Gordon, 2019; Rozengurt, 2023; Rozengurt, 2017; Tseng, 2021) [12, 33–37]
**B. BA (Hz)**		
**1. Theta (3–8)**	involving 3.6–7.5 Hz, 3–7 Hz, 4–7 Hz, 4–8 Hz	(Becerra, 2012) [9], (Brandmeyer, 2020; Shtoots, 2021) [41, 95], (Vernon, 2003; Wang, 2013) [30, 98], (Chikhi, 2024; Eschmann, 2020; Eschmann, 2021; Enriquez-Geppert, 2014; Marcos-Martínez, 2023; Rozengurt, 2017; Smit, 2023) [11, 25–27, 33, 67, 69]
**2. Alpha (7–13)**	involving 7–13 Hz, 8–12 Hz, 8–13 Hz	(Diotaiuti, 2024; Li, 2023; Paban, 2024) [36, 90, 92], (Chen, 2023; Domingos, 2020; Domingos, 2021a; Domingos, 2021b; Hsueh, 2016; Hsueh, 2012; Nan, 2012; Pei, 2018; Shen, 2023; Wei, 2017; Zhou, 2024) [16, 17, 28, 29, 44, 52, 74–77, 79], (Nawaz, 2023) [42]
●**UA (10-13.5)**	involving IAF to IAF + 2 Hz, 10–12 Hz, 10.2–12.2 Hz, 10–13.5 Hz	(Escolano, 2014; Escolano, 2012; Gordon, 2019; Hanslmayr, 2005; Uslu, 2023; Zoefel, 2011) [18, 34, 43, 72, 85, 86], (Chikhi, 2024; Chikhi, 2023; Farraj, 2024; Guez, 2014; Lecomte, 2011) [26, 37, 65, 73, 89], (Alekseeva, 2012) [84], (Naas, 2019) [68]
**3. Beta (12–22)**	involving 12–20 Hz, 15–22 Hz	(Farnia, 2017; Keizer, 2010a; Staufenbiel, 2014) [87, 88, 96], (Rozengurt, 2017) [33]
●**SMR (11.6–16)**	involving 11.6–16, 12–15 Hz, 13–15 Hz	(Berner, 2006) [14], (Campos da Paz, 2018; Fritson, 2007; Hsueh, 2012; Kober, 2020; Kober, 2017; Kober, 2015; Nazer, 2018; Van Eijk, 2017; Vernon, 2003) [10, 15, 19, 28, 66, 71, 80, 91, 98], (Guez, 2014) [65]
●**LB (15–18)**	involving 15–18 Hz	(Shtoots, 2021) [95]
**4. Gamma (30–45)**	involving 30–45 Hz, 35–45 Hz, 36–44 Hz, 40–43 Hz	(Salari, 2012, 2014) [32, 94], (Andrade, 2024) [78], (Keizer, 2010a; Keizer, 2010b; Khodakarami, 2020; Staufenbiel, 2014) [24, 31, 88, 96], (Kober, 2017) [80]
**C. BA mixed training**		
**1. Training of BAR**	involving LA (7–9.5 Hz)/UA (9.5–12 Hz), Theta (4–7 Hz)/Alpha (8–12 Hz), Theta (4–8 Hz)/LB (14–18 Hz), Beta (15–22 Hz)/Theta (4–8 Hz)	(Farnia, 2017) [87], (Xiong, 2014) [13], (Rozengurt, 2023; Tseng, 2021) [12, 35], (Rozengurt, 2023) [35]
**2. BA + the others**	involving sham + WMT, Alpha (8–13 Hz) + WMT, Alpha (8–12 Hz) + Theta (4–8 Hz), UA (10–12 Hz) + WMT, UA (10–12 Hz) + PC	(Parsons, 2021) [93], (Parsons, 2021) [93], (Vekety, 2022) [97], (Gordon, 2019) [34], (Gordon, 2019) [34]

Abbreviations: *AC*, active control; *ADL*, activities of daily living; *BA*, brain activity; *BAR*, brain-activity ratios; *EEG*, electroencephalogram; IAF, individual alpha frequency; *LA*, lower alpha; *LB*, lower beta; *NFT*, neurofeedback training; *PC*, passive control; *SC*, silent control; *SMR*, sensorimotor rhythm; *UA*, upper alpha; *WMT*, working memory training

### Results of the Network Meta-Analysis (NMA) on Working Memory (WM)

**Fig. 2:**
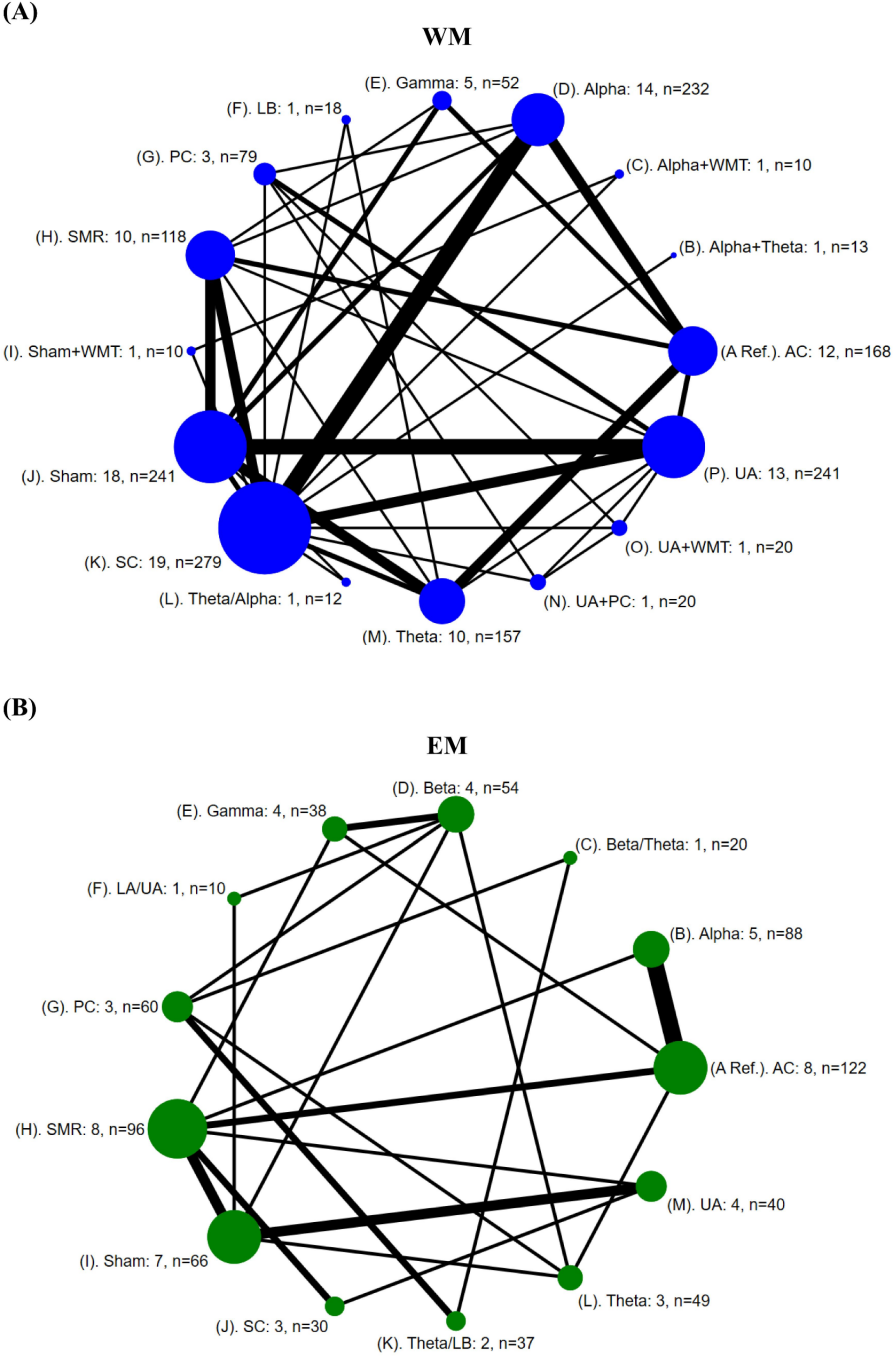
Network geometry for EEG-NFT on WM (**A**) and EM (**B**) in healthy participants. Each node indicates a particular intervention and is weighted according to the number of studies. Each edge (line connecting the nodes) is weighted according to the number of participants and directly compares the interventions it connects. Each English number respectively shows a series of information (an intervention: number of studies, n = number of participants). *AC*, active control; *LA*, lower alpha; *LB*, low beta; *PC*, passive control; *Ref.*, reference; *SC*, silent control; *SMR*, sensorimotor rhythm; *UA*, upper alpha; *WMT*, working memory training

Figure 2A shows NMA for EEG-based NFT on WM. Fifty trials (1670 participants) with a reference AC compared to 15 interventions (alpha + theta, alpha + WMT, alpha, gamma, LB, PC, SMR, sham + WMT, sham, SC, theta/alpha, theta, UA + PC, UA + WMT, and UA) examined the impact of the interventions.

**Fig. 3:**
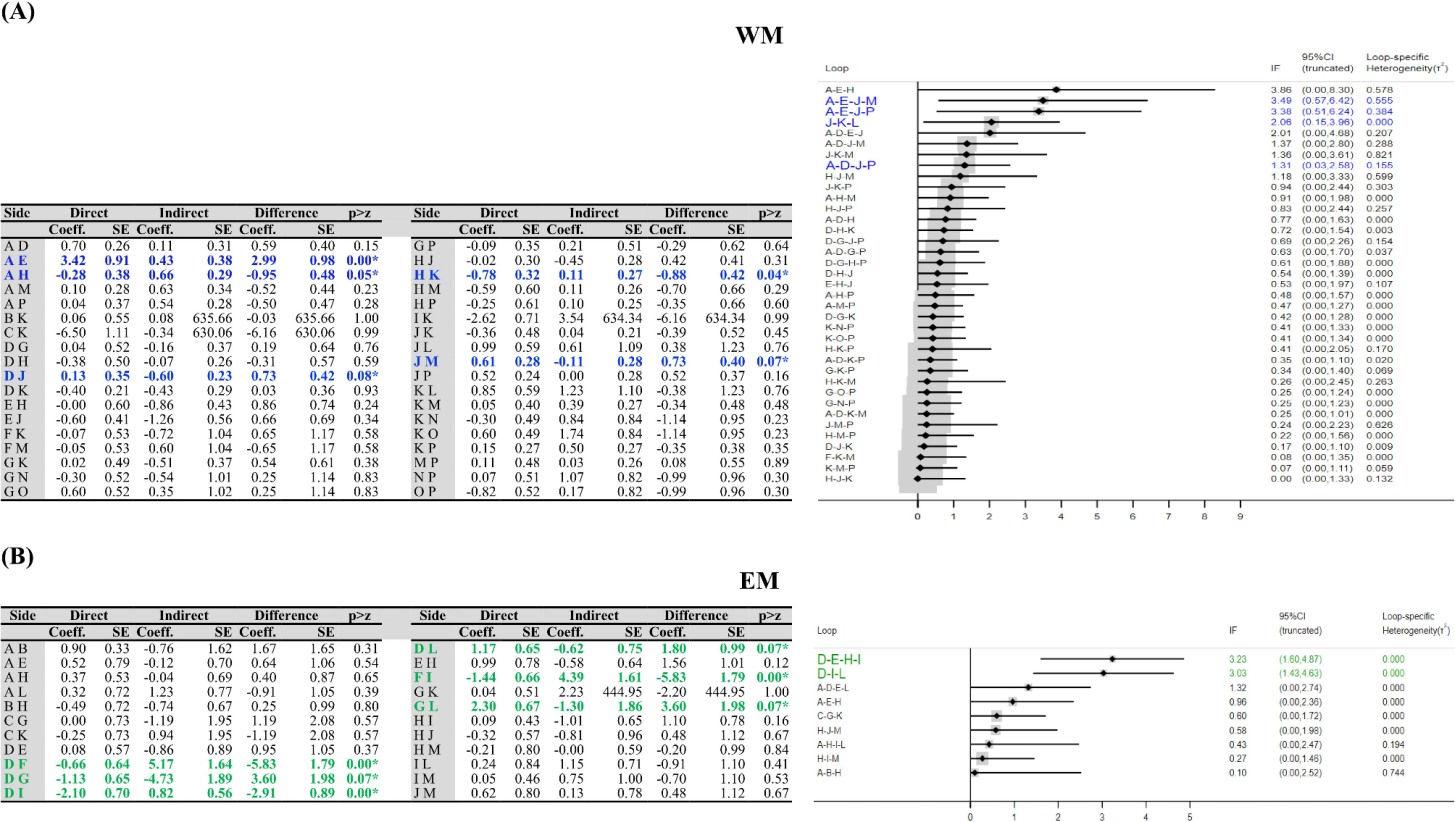
Inconsistency test through side-splitting models and loop-specific approach for EEG-NFT on WM (**A**) and EM (**B**). [WM (A = AC, B = alpha + theta, C = alpha + WMT, D = alpha, E = gamma, F = LB, G = PC, H = SMR, I = sham + WMT, J = sham, K = SC, L = theta/alpha, M = theta, N = UA + PC, O = UA + WMT, and P = UA); EM (A = AC, B = alpha, C = beta/theta, D = beta, E = gamma, F = LA/UA, G = PC, H = SMR, I = sham, J = SC, K = theta/LB, L = theta, and M = UA)]. *Coeff.*, coefficient; *CI*, confidence interval; *SE*, standard error

Figure 3A shows the tests of inconsistency with the loop-inconsistency models between direct and indirect evidences. Prior to evaluate local approach, global inconsistence was performed and showed no significant inconsistency (χ^2^(22) = 30.89, *p* = 0.10). However, side-splitting models showed great inconsistency between the AC-gamma (*p* < 0.001), AC-SMR (*p* = 0.05), alpha-sham (*p* = 0.08), SMR-SC (*p* = 0.04), and sham-theta (*p* = 0.07). Additionally, a loop-specific approach in the AC-gamma-sham-theta, AC-gamma-sham-UA, sham-SC-theta/alpha, and AC-alpha-sham-UA showing the direct and indirect effect estimated of one of the training comparisons within this closed loop was significant difference from one another because 95% CI range in loop does not include across zero.

**Fig. 4:**
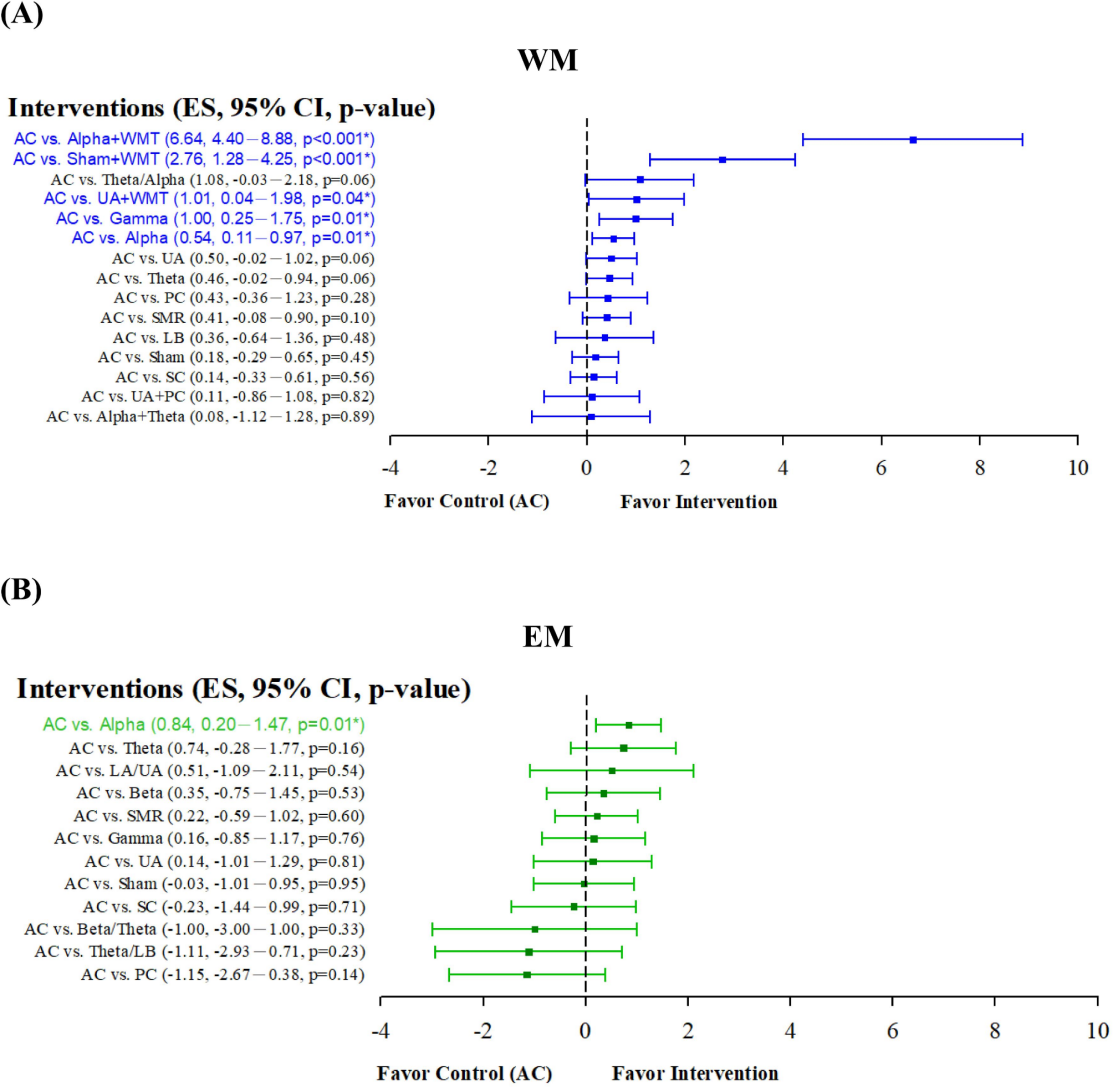
Consistency assessment through forest plots showing a pairwise comparison of AC versus included interventions in terms of EEG-NFT on WM (**A**) and EM (**B**). *CI*, confidence interval; *ES*, effect size

**Fig. 5:**
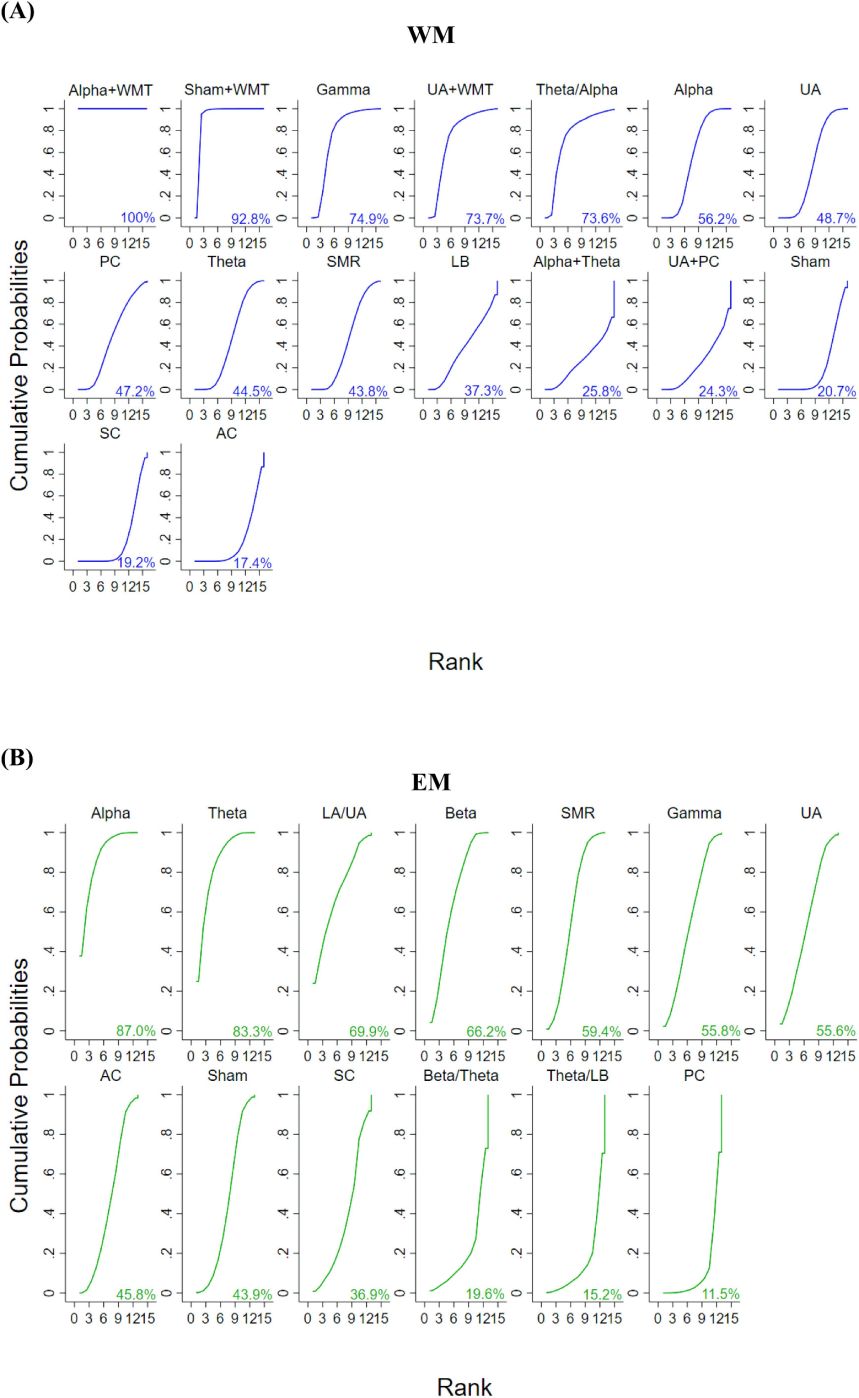
Efficacy ranking through SUCRAs respectively exhibiting EEG-NFT on WM (**A**) and EM (**B**). Ranking indicates the probability to be the best treatment, the second best, and so on. A larger SUCRA score indicates a more effective intervention

Figure 4A shows the consistency assessment referring the potentially efficacy of EEG-based brain activity NFT on WM in the forest plot of pairwise comparison. The results showed that a reference (AC) respectively compared to alpha + WMT, sham + WMT, UA + WMT, gamma, and alpha showed significant difference. Specially, AC versus alpha + WMT, sham + WMT, and UA + WMT respectively exhibited no any inconsistency between the direct and indirect effect from one another in Fig. 3A, indicating that the training efficacy of these interventions was accepted in the consistency assessment. Meanwhile, the results of SUCRA values showed that alpha + WMT (100%) was the most effective intervention to increase WM presented in Fig. 5A.

Figure 5A also shows the SUCRA value, indicating that EEG-NFT has less impact on WM in the control groups. The results showed the rank of control groups based on the effectiveness of interventions in evoking EEG-NFT on WM, from the largest to the smallest (PC = 47.2%, sham = 20.7%, SC = 19.2%, and AC = 17.4%).

**Fig. 6:**
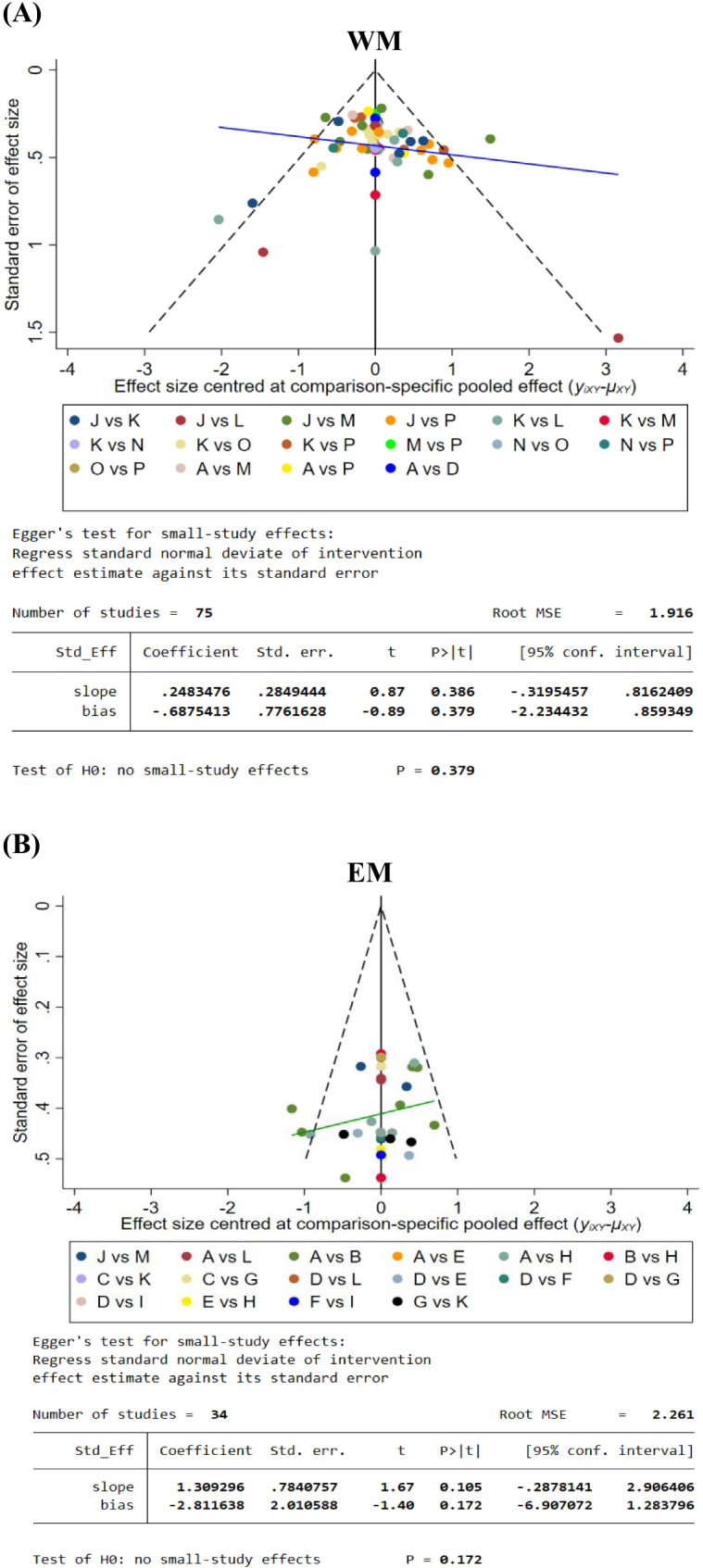
Publication bias and egger’s test for the EEG-NFT on WM (**A**) and EM (**B**). The black line represents the null hypothesis that the study-specific effect sizes do not differ from the respective comparison-specific pooled effect estimates. The blue and green lines respectively represent the regression line on WM and EM. The different colors correspond to different comparisons. [WM (A = AC, B = alpha + theta, C = alpha + WMT, D = alpha, E = gamma, F = LB, G = PC, H = SMR, I = sham + WMT, J = sham, K = SC, L = theta/alpha, M = theta, N = UA + PC, O = UA + WMT, and P = UA); EM (A = AC, B = alpha, C = beta/theta, D = beta, E = gamma, F = LA/UA, G = PC, H = SMR, I = sham, J = SC, K = theta/LB, L = theta, and M = UA)]. *conf.*, confidence; *MSE*, mean standard error; *Std_eff*, standard effects; *Std. err.*, standard error

Figure 6A shows the comparison-adjusted funnel plots and Egger’s tests on WM. The results exhibited a relatively symmetrical distribution, indicating no publication bias in EEG-NFT on WM (*p* = 0.38).

In the supplementary results, fig. S1 presents the NMA results for WM with one study excluded for sensitivity analysis [31]. The node-splitting models following the acceptance of the consistency model for alpha + WMT (Tables S1 & S2), SUCRA values for the top EEG-NFT intervention as well as the control group levels (Table S3), and the publication bias results (Fig. S2) are consistent with those from the non-excluded results. These findings confirm the robustness of WM.

## Results of the Network Meta-Analysis (NMA) on Episodic Memory (EM)

Figure 2B shows NMA for EEG-based NFT on EM. Twelve-four trials (710 participants) with a reference AC compared to 12 interventions (alpha, beta/theta, beta, gamma, LA/UA, PC, SMR, sham, SC, theta/LB, theta, and UA) examined the impact of the interventions.

Figure 3B shows the tests of inconsistency with the loop-inconsistency models between direct and indirect evidences. Prior to evaluate local approach, global inconsistence was performed and showed no significant inconsistency (χ^2^(10) = 13.48, *p* = 0.19). However, side-splitting models showed great inconsistency between the beta-LA/UA (*p* < 0.001), beta-PC (*p* = 0.07), beta-sham (*p* < 0.001), beta-theta (*p* = 0.07), LA/UA-sham (*p* < 0.001), and PC-theta (*p* = 0.07). Additionally, a loop-specific approach in the beta-gamma-SMR-sham and beta-sham-theta showing the direct and indirect effect estimated of one of the training comparisons within this closed loop was significant difference from one another due to 95% CI not included across zero.

Figure 4B shows the consistency assessment referring the potentially efficacy of brain activities on EM in the forest plot of pairwise comparison. The results showed that a reference (AC) compared to alpha was significant difference. Specially, AC versus NFT of alpha activity exhibited no any inconsistency between the direct and indirect effect from one another in Fig. 3B, indicating that the efficacy of alpha NFT was accepted in the consistency assessment. Meanwhile, the results of SUCRA values showed that alpha (87.0%) was the most effective intervention to increase EM presented in Fig. 5B.

Figure 5B also shows the SUCRA value, indicating EEG-NFT has less impact on EM in the control groups. The results showed the rank of control groups based on the effectiveness of interventions in evoking EEG-NFT on EM, from the largest to the smallest (AC = 45.8%, sham = 43.9%, SC = 36.9%, and PC = 11.5%).

Figure 6B shows the comparison-adjusted funnel plots and Egger’s tests on EM. The results exhibited a relatively symmetrical distribution, indicating no publication bias in EEG-NFT on EM (*p* = 0.17).

In the supplementary results, fig. S3 presents the NMA results for EM with one study excluded for sensitivity analysis [14]. The node-splitting models following the acceptance of the consistency model for alpha NFT (Tables S4 & S5), SUCRA values for the top EEG-NFT intervention as well as the control group levels (Table S6), and the publication bias results (Fig. S4) are consistent with those from the non-excluded results. These findings confirm the robustness of EM.

## Discussion

Sixty clinical trials were included in this study, involving 1995 participants. The network of EEG-NFT on WM included 50 trials (1670 participants) with a reference AC compared to 15 interventions, and the network of EM included 24 trials (710 participants) with a reference AC compared to 12 interventions. The results of EEG-NFT on WM showed no significant global inconsistency. In local inconsistency evaluations, there was no significant influence related to AC compared to alpha + WMT, suggesting that significant differences were observed and accepted in pairwise consistency evaluations. The SUCRA values showed that the best efficacy was achieved with the combination of alpha and WMT in EEG-NFT on WM. Additionally, EEG-NFT on EM, global inconsistency evaluations exhibited no significant difference, and local inconsistency evaluations represented no significant differences related to AC versus alpha NFT, suggesting that significant differences were observed and accepted in pairwise consistency evaluations. The SUCRA evaluation showed that NFT of alpha activity had the best efficacy in increasing EM. Regarding the control groups, AC or SC evoked less brain activity affecting WM. Compared to EM, PC or SC has less effect on EEG-NFT for EM. The publication bias for EEG-NFT on WM and EM respectively showed a symmetrical funnel with no significant effect. Sensitivity analysis confirms the robustness of the results for WM and EM. Taken together, EEG-NFT related to alpha activity appears to be the most effective for enhancing memory in healthy participants, particularly in EM.

Experimental designs among the variance in sample size, differences in memory evaluations, and heterogeneity in the training protocols of EEG-NFT are believed to impact the outcomes of local inconsistency. The results may be influenced by the fact that this study is a holistic NMA, primarily investigating the best efficacy of EEG-NFT on memory, integrating data from multiple sources. For instance, BDST and FDST involve different strategies or representations depending on the direction of recall [46]. A functional magnetic resonance imaging study suggests that the MRT and N-back tasks activate different brain areas [47]. While these tasks are used to assess WM, there are some differences among them, which may affect WM results. Therefore, the different effect sizes of EEG-NFT and the heterogeneous cognitive tasks for WM and EM between direct and indirect evidence reflect a substantial variation and heterogeneity in the local inconsistency results. Our results suggest that a more standardized approach to memory evaluation and EEG-NFT training protocols across studies is necessary to reduce heterogeneity and improve the reliability and validity of the findings.

For participant characteristics (e.g., age, gender, or educational attainment), these factors may also impact the results due to the heterogeneity of local inconsistencies. For instance, alpha activity changes across different age stages. Children and older adults tend to have an alpha peak at frequencies below 10 Hz, whereas young adults show a peak ≥10 Hz [48]. Regarding gender, previous studies have reported differences in beta and theta activity between males and females across different brain regions during cognitive tasks [49, 50]. Additionally, individuals with higher educational attainment tend to exhibit better cognitive performance compared to those with lower educational levels [51]. These reports suggest that EEG-NFT research may also need to consider participant selection to reduce heterogeneity. Hence, variance in participant characteristics may contribute to the heterogeneity of local inconsistencies in the present study.

The efficacy of EEG-NFT on WM seems to be due to the combination of alpha activity and cognitive training. In the previous studies, NFT of alpha activity exhibited significant enhancement of WM [17, 52]. Some articles have found little memory improvement throughout alpha NFT [48, 53]. Specifically, a recent study suggests that alpha NFT may not be beneficial for WM enhancement [54]. These controversial results may arise from the lack of combined training for WM. Indeed, SUCRA results showed that the combined training (i.e., alpha NFT combined WMT (SUCRA = 100%) or UA NFT combined WMT (SUCRA = 73.7%) is beneficial for WM. Compared with single alpha and UA NFTs, SUCRA values of 56.2% and 48.7%, respectively, exhibited a medium efficacy on WM in the present study. This may explain the controversial EEG-NFT on WM results. Therefore, alpha activity NFT combined with cognitive training may have a greater influence on EEG-NFT for WM, according to a previous meta-analysis [55].

The efficacy of EEG-NFT in the alpha frequency activity treatment protocols is believed to impact the enhancement of EM. EM formation involves and begins with the perceptual data of an experienced episodic memory process in sensory brain regions [56]. Therein, ongoing alpha-band oscillations play a crucial role in perceptual experience, being related to the impact of perceptual learning on EM ability and capacity [57, 58]. In the present study, the SUCRA results exhibited alpha activity showing a strong efficacy of EEG-NFT on EM. This phenomenon suggests that alpha activity may be an important aspect of brain activity for EM occurrence. The combined training of the included studies for EM shows little sample size in this study; therefore, alpha NFT combined with others for EM requires more powerful studies.

Why the NFT of alpha activity is important for enhancing memory? Several lines of evidence offer plausible explanations for this phenomenon. Event-related potential analysis has shown that the amplitude of alpha activity is enhanced during the retention phase of both WM [59] and EM [60]. It has been proposed that alpha oscillations during the memory retention period are essential for the network activity that sustains the neuronal representations of memory items [61, 62]. The suppression of alpha activity during memory retrieval partially reflects the termination of the memory process [63]. Furthermore, alpha activity plays an active role in inhibiting task-irrelevant processes and regulating the timing of cortical processing [61]. Additionally, neuroimaging studies using the selective reminding task with free and cued recall demonstrate that the suppression of alpha activity contributes to short-term EM dysfunction [64]. These findings suggest that alpha activity may be important for the engagement of WM and EM processes. In the present study, alpha activity also demonstrates significantly greater efficiency in improving WM and EM compared to the control group, as shown in the consistency results.

Regarding the intensity and dose of NFT, considerable efforts have been made to optimize training parameters. A previous meta-analysis on the effects of alpha and theta NFTs on memory demonstrated that a single session lasting more than 20 min can significantly improve working memory (WM) [5, 7]; however, the SUCRA values for NFT on WM indicate that alpha (56.2%) and theta (44.5%) have only moderate efficacy. One possible reason is that the meta-analysis accounts for the overall effect across multiple studies, some of which report no significant enhancement in WM. Additionally, a recent study [54] suggests that cognitive training is more effective for WM improvement than EEG-NFT, which aligns with our SUCRA analysis showing that training involving WMT achieves an efficacy of > 70% for WM. In contrast, for EM, a meta-analysis of alpha and theta NFTs with more than 10 sessions of at least 20 min per session shows a greater impact, with SUCRA values indicating high efficacy (alpha: 87.0%, theta: 83.3%). Based on these SUCRA rankings, an optimized training parameters has been proposed: for WM, a combination of NFT and WMT with a single session lasting more than 20 min; for EM, NFT alone with more than 10 sessions, each lasting over 20 min.

The different control groups have raised an issue about the effect of EEG-NFT on memory. A previous study suggests that there is minimal control/placebo influence in an NFT route and a significant contribution of NFT to memory enhancement [5]. Indeed, in this study, the SUCRA value shows that the AC of control groups has less impact on NFT with respect to WM. This suggests that WM may be only partly affected by NFT. Conversely, cognitive training (i.e., WMT) has a greater impact on WM enhancement. Our results show that brain activity involving WMT has an efficacy of over 70%, specifically, alpha + WMT at 100% and UA + WMT at 73.7%. Additionally, the sham control, which involves no brain activity, combined with WMT, represents an efficacy of 92.8%. These results suggest that WMT contributes more significantly to WM improvement than EEG-NFT, as demonstrated in a recent study [54]. On the other hand, the PC group shows less efficiency in applying NFT to EM, despite involving cognitive-like training, such as target search of the same letter as word-paired-like training [34], recall of movie plots as cognitive-perceptual activity [33], or Sudoku game gaps filled with the corresponding numbers as cognitive exercise [35]. These findings suggest that PC may involve cognitive training and imply that EEG-based brain activity NFT has a greater impact on EM enhancement than cognitive exercising training.

Additionally, the SC may serve as a better control for experimental intervention, whereas the control groups of EEG-NFT with different intervention types (i.e., SC, sham, AC, or PC) cause controversial results. For instance, some studies comparing sham to the EEG-NFT on memory exhibited significant differences [65–67]. Compared with some studies, WM [15, 44, 68, 69] and EM [14] exhibited no difference between sham and NFT throughout intervention. Likewise, studies comparing AC to EEG-NFT showed significant improvements in WM and EM throughout intervention [16, 17, 27, 29], whereas some articles did not [11, 18, 19]. In the previous study, the efficacy of AC differs from the sham condition in terms of task performance [70]. This phenomenon may influence the results, with varying degrees of difference. On the other hand, the comparison between the SC and EEG-NFT on memory also represents the different results (except for one study not comparing the two groups [13]) but only 4 of 18 studies (22.2%) reported no significant enhancement in memory with EEG-NFT [34, 71–73]. Specifically, the SUCRA values of WM and EM in the SC represented efficacy ranging from 19 to 36%, which is similar to the 22.2% efficacy. Importantly, the comparison of control groups based on SUCRA efficacy shows that the SC for both WM and EM has the same ranking, placing it at the second-lowest level. These findings suggest that the SUCRA results are reliable and imply that the SC, with less brain activity influencing memory compared to other interventions, may be a better option as a control group for EEG-NFT.

Interestingly, among the included studies comparing the SC and EEG-NFT, those showing no significant difference [34, 71–73] all involved MRT evaluations. In contrast, all studies using n-back evaluation [42, 74–77], except for one study that did not compare the groups [13], showed significant WM improvement with EEG-NFT compared to SC. Meanwhile, the digit-span task in the included studies also exhibited a significant difference in all studies comparing the SC to EEG-NFT [16, 17, 19, 28, 29, 52, 66, 68, 78–80]. These findings may arise from the fact that MRT involves brain activation in bilateral areas, whereas the n-back task primarily requires activation in the unilateral right brain area [47]. Specifically, the brain activation patterns of the digit-span task are similar to those of the n-back task [81]. These findings suggest that the mechanisms underlying WM in MRT differ from those in digit-span and n-back tasks, which may contribute to divergent results in EEG-NFT studies on memory.

Taken together, the current study provides insight into the most effective brain activity on both WM and EM through EEG-NFT. NFT of alpha activity combined with cognitive training is beneficial for WM. Compared to EM, alpha activity is primarily effective in enhancing EM through EEG-NFT. As for control groups, the SC group seems to be a better option for comparison with EEG-NFT on both WM and EM than the others. Additionally, the variety of WM tasks may be a key factor contributing to the divergent results of EEG-NFT on memory in healthy participants.

### Strength and weakness

The strengths of this study include the use of PROSPERO to pre-register the protocol for this review and the adherence to PRISMA guidelines in preparing and reporting this systematic review and network meta-analysis [82]. Importantly, it is the first study to exclusively investigate the effects of a variety of brain activities and control protocols on memory through an NMA. Consequently, a comprehensively rigorous review and credible results exist in the present study. This study highlights key points that could improve WM and EM in healthy participants using EEG-NFT, especially in alpha activity.

Even if comprehensive and based on the PICOS framework with accurate search strategies, several potential weaknesses still exist in this study. Some potentially relevant studies of interest may have been missed from master’s theses or doctoral dissertations because they were not published yet. Additionally, studies written in non-English languages were excluded. Although excluding non-English studies from evidence syntheses does not affect conclusions [83], this bias may still influence the results of the evidence synthesis. Besides, for the six studies that were missing data from our screening results, we contacted the corresponding authors via email. Only two authors replied to our request and provided data for the quantitative synthesis of this study. In summary, the conclusions are based on the best evidence synthesis available, but they still need to be interpreted carefully given these weaknesses.

### Caution

There are several points that still need to be interpreted with caution in this study. Firstly, training parameters (i.e., session and duration) were not classified for analysis. We investigated which brain activity is the most effective for WM and EM, given that a wide range of brain activity (i.e., theta, alpha, UA, beta, gamma, and so on) have been reported to have an efficacy of enhancing memory according to EEG-NFT studies. Secondly, the effects of EEG-NFT on memory for alpha and theta activities have been evaluated through systematic reviews assessing the quality of included studies [5, 7]. However, brain activities (i.e., beta and gamma) related to bias assessment and systematic review have not been reported until now. Thirdly, brain activity combined with others, such as alpha + WMT, UA + WMT, or alpha + theta, involving a small number of studies for WM was included in this NMA. Finally, inconsistency analysis using a side-by-side splitting model shows a significant difference in WM and EM. Taken together, these factors may have influenced the results and require caution.

## Conclusion

Our results obtained through the NMA of the latest methods comparing multiple interventions, demonstrate significant WM enhancement through alpha activity combined with cognitive training, and EM improvement in healthy participants using alpha activity NFT. These findings demonstrate that alpha activity (7–13 Hz) may be a crucial factor impacting memory performance. Compared with the control, the SC appears to cause less interference with brain activity and have a smaller impact on memory than the other control groups. Further investigation is needed to explore how various WM tasks influence specific brain areas, particularly in combination with EEG or functional magnetic resonance imaging studies. Additionally, more robust studies are required to examine the impacts of NFT combined with other training methods on memory and cognition in the future.

## Electronic supplementary material

Below is the link to the electronic supplementary material.

Supplementary Material 1

## Data Availability

No datasets were generated or analysed during the current study.
