# Supplementary material for: Comparative effectiveness of electroencephalogram-neurofeedback training of 3–45 frequency band on memory in healthy population: a network meta-analysis with systematic literature search
Source: J Neuroeng Rehabil. 2025 Apr 24;22:94. doi: 10.1186/s12984-025-01634-8 (PMC12020070; doi:10.1186/s12984-025-01634-8)
Supplement: Supplementary file 1 — Supplementary Material 1 [file 12984_2025_1634_MOESM1_ESM.docx]

**Comparative effectiveness of electroencephalogram-neurofeedback training of 3-45 frequency band on memory in healthy population: A network meta-analysis with systematic literature search**

**Wen-Hsiu Yeh^1^*, Ya-Ju Ju^2,3^, Fu-Zen Shaw^4,5^, Yu-Ting Liu^6,7^**

^1^Department of Gerontological Health Care, Central Taiwan University of Science and Technology, Taichung City 406, Taiwan

^2^Teaching and Research Center, Kaohsiung Municipal Siaogang Hospital, Kaohsiung Medical University Hospital, Kaohsiung Medical University, Kaohsiung 812, Taiwan

^3^Department of Physical Therapy, College of Health Care, China Medical University, Taichung 406, Taiwan.

^4^Department of Psychology, National Cheng Kung University, Tainan 701, Taiwan

^5^Mind Research and Imaging Center, National Cheng Kung University, Tainan, 701, Taiwan

^6^Department of Medical Science Industries, Chang Jung Christian University, Tainan 711, Taiwan

^7^Bachelor Degree Program in Medical Sociology and Health Care, Chang Jung Christian University, Tainan 711, Taiwan

**^*^Corresponding author:**

Wen-Hsiu Yeh, PhD

Department of Gerontological Health Care

Central Taiwan University of Science and Technology

No.666, Buzih Road, Beitun District, Taichung City 406053, Taiwan (R.O.C.)

Tel: +886-22391647 ext 6254 / E-mail: wenhsiu0810@gmail.com

**Supplementary results**

Fig. S1 shows NMA for EEG-based NFT on WM, with the smallest-sample study removed from a dataset (Khodakarami et al., 2020). Forty-nine trials (1662participants) with a reference AC compared to 15 interventions (alpha+theta, alpha+WMT, alpha, gamma, LB, PC, SMR, sham+WMT, sham, SC, theta/alpha, theta, UA+PC, UA+WMT, and UA) examined the impact of the interventions.


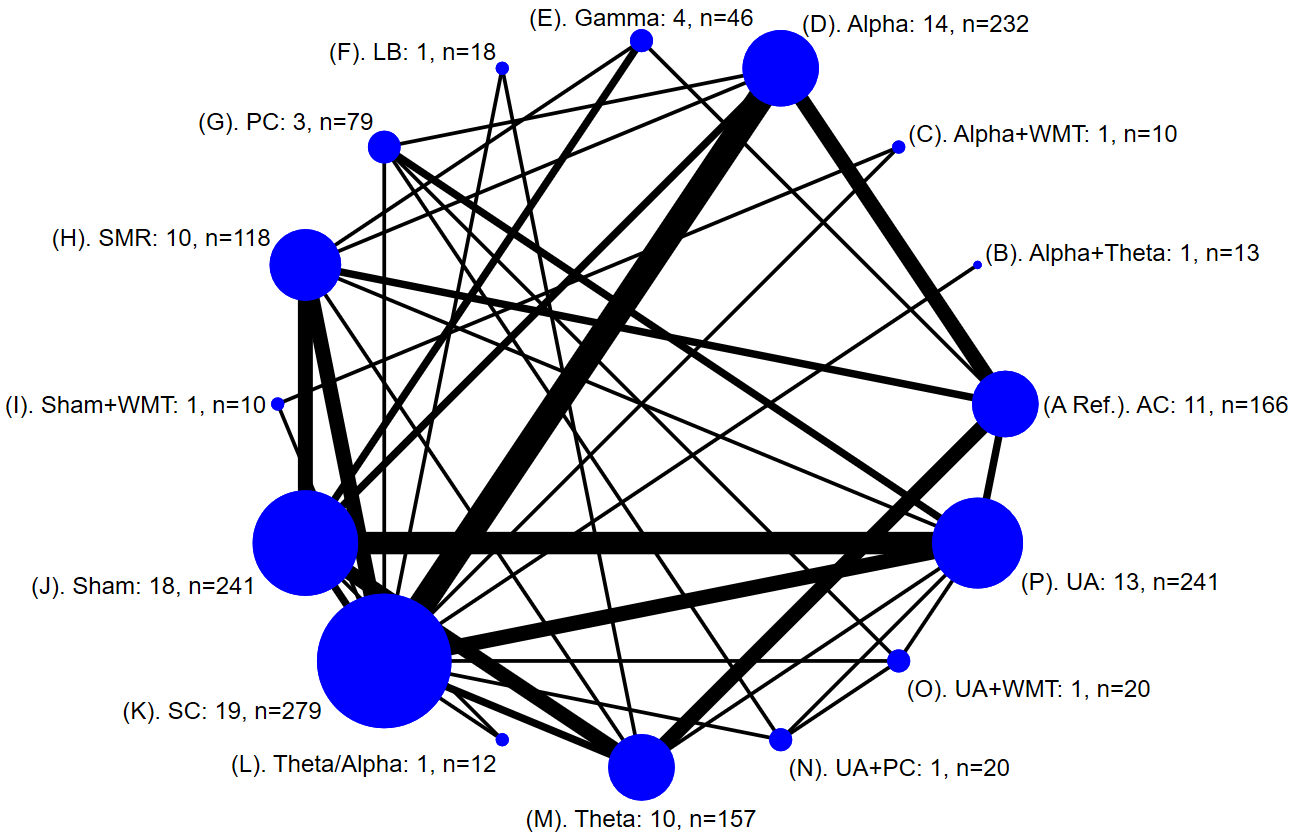
**Working memory (WM)**

**Fig. S1 Network geometry for EEG-NFT on WM in healthy participants.** Each node indicates a particular intervention and is weighted according to the number of studies. Each edge (line connecting the nodes) is weighted according to the number of participants and directly compares the interventions it connects. Each English number respectively shows a series of information (an intervention: number of studies, n=number of participants). *AC,* active control; *LA,* lower alpha; *LB,* low beta; *PC,* passive control; *Ref.,* reference; *SC,* silent control; *SMR,* sensorimotor rhythm; *UA,* upper alpha; *WMT,* working memory training.

Table S1 shows the inconsistency tests using loop-inconsistency models to analyse direct and indirect evidence on WM, with the smallest-sample study removed from a dataset (Khodakarami et al., 2020). Global inconsistence showed the analysed results (χ^2^(22)=36.44, p=0.03). Side-splitting models showed great inconsistency between the AC-gamma (p<0.001), AC-SMR (p=0.06), alpha-sham (p=0.07), SMR-SC (p=0.03), and sham-theta (p=0.08).

**Table S1 The inconsistency tests using loop-inconsistency models on WM**

| **Side** | **Direct** | | **Indirect** | | **Difference** | | **p>z** |  | **Side** | **Direct** | | **Indirect** | | **Difference** | | **p>z** |
| --- | --- | --- | --- | --- | --- | --- | --- | --- | --- | --- | --- | --- | --- | --- | --- | --- |
|  | **Coeff.** | **SE** | **Coeff.** | **SE** | **Coeff.** | **SE** |  |  |  | **Coeff.** | **SE** | **Coeff.** | **SE** | **Coeff.** | **SE** |  |
| A D | 0.69 | 0.26 | 0.05 | 0.31 | 0.64 | 0.40 | 0.11 |  | G P | -0.09 | 0.34 | 0.20 | 0.51 | -0.29 | 0.62 | 0.64 |
| **A E** | **6.51** | **1.58** | **0.42** | **0.38** | **6.09** | **1.62** | **0.00** |  | H J | -0.02 | 0.30 | -0.45 | 0.28 | 0.43 | 0.41 | 0.30 |
| **A H** | **-0.28** | **0.37** | **0.62** | **0.29** | **-0.91** | **0.48** | **0.06** |  | **H K** | **-0.78** | **0.32** | **0.12** | **0.27** | **-0.90** | **0.42** | **0.03** |
| A M | 0.10 | 0.28 | 0.58 | 0.34 | -0.48 | 0.44 | 0.27 |  | H M | -0.59 | 0.60 | 0.12 | 0.26 | -0.72 | 0.66 | 0.28 |
| A P | 0.04 | 0.37 | 0.51 | 0.28 | -0.47 | 0.46 | 0.31 |  | H P | -0.25 | 0.60 | 0.11 | 0.25 | -0.36 | 0.65 | 0.59 |
| B K | 0.06 | 0.55 | 0.04 | 634.12 | 0.02 | 634.12 | 1.00 |  | I K | -2.62 | 0.71 | 3.49 | 634.30 | -6.11 | 634.30 | 0.99 |
| C K | -6.50 | 1.11 | -0.39 | 631.62 | -6.11 | 631.62 | 0.99 |  | J K | -0.35 | 0.48 | 0.05 | 0.21 | -0.40 | 0.52 | 0.44 |
| D G | 0.04 | 0.52 | -0.16 | 0.37 | 0.20 | 0.63 | 0.75 |  | J L | 0.99 | 0.59 | 0.64 | 1.08 | 0.35 | 1.22 | 0.78 |
| D H | -0.38 | 0.50 | -0.08 | 0.26 | -0.29 | 0.56 | 0.60 |  | **J M** | **0.61** | **0.28** | **-0.09** | **0.28** | **0.70** | **0.40** | **0.08** |
| **D J** | **0.13** | **0.35** | **-0.63** | **0.23** | **0.75** | **0.42** | **0.07** |  | J P | 0.52 | 0.24 | 0.02 | 0.27 | 0.50 | 0.36 | 0.17 |
| D K | -0.40 | 0.21 | -0.44 | 0.28 | 0.04 | 0.35 | 0.90 |  | K L | 0.85 | 0.59 | 1.20 | 1.09 | -0.35 | 1.22 | 0.78 |
| E H | 0.00 | 0.60 | -0.76 | 0.45 | 0.76 | 0.75 | 0.31 |  | K M | 0.05 | 0.40 | 0.39 | 0.27 | -0.34 | 0.48 | 0.47 |
| E J | -0.60 | 0.41 | -1.09 | 0.62 | 0.49 | 0.74 | 0.51 |  | K N | -0.30 | 0.48 | 0.84 | 0.84 | -1.14 | 0.94 | 0.22 |
| F K | -0.07 | 0.53 | -0.72 | 1.03 | 0.66 | 1.16 | 0.57 |  | K O | 0.60 | 0.48 | 1.74 | 0.84 | -1.14 | 0.94 | 0.22 |
| F M | -0.05 | 0.53 | 0.60 | 1.03 | -0.66 | 1.16 | 0.57 |  | K P | 0.15 | 0.27 | 0.50 | 0.26 | -0.35 | 0.37 | 0.35 |
| G K | 0.02 | 0.49 | -0.51 | 0.37 | 0.54 | 0.60 | 0.37 |  | M P | 0.11 | 0.48 | 0.03 | 0.26 | 0.08 | 0.54 | 0.88 |
| G N | -0.30 | 0.52 | -0.55 | 1.01 | 0.25 | 1.13 | 0.83 |  | N P | 0.07 | 0.51 | 1.06 | 0.81 | -0.99 | 0.95 | 0.30 |
| G O | 0.60 | 0.52 | 0.35 | 1.01 | 0.25 | 1.13 | 0.83 |  | O P | -0.82 | 0.51 | 0.16 | 0.81 | -0.99 | 0.95 | 0.30 |
| **Notes:** A=AC, B=alpha+theta, C= alpha+WMT, D=alpha, E=gamma, F=LB, G=PC, H=SMR, I=sham+WMT, J=sham, K=SC, L=theta/alpha, M=theta, N=UA+PC, O=UA+WMT, and P=UA.  **Abbreviations:** *Coeff.,* coefficient; *CI,* confidence interval; *SE,* standard error. | | | | | | | | | | | | | | | | |

Table S2 displays the consistency assessment showing a pairwise comparison of AC versus included interventions in terms of EEG-NFT on WM, with the smallest-sample study removed from a dataset (Khodakarami et al., 2020). The results showed that a reference (AC) respectively compared to alpha+WMT and sham+WMT showed significant difference. Specially, AC(A) versus alpha+WMT(C) and sham+WMT(I) respectively exhibited no any inconsistency between the direct and indirect effect from one another in Table S1, indicating that the training efficacy of these interventions was accepted in the consistency assessment.

**Table S2 The consistency assessment on WM**

| **Rank of ES** | **Interventions** | **(ES, 95% CI, p-value)** | **Rank of ES** | **Interventions** | **(ES, 95% CI, p-value)** |
| --- | --- | --- | --- | --- | --- |
| **1** | **AC vs. Alpha+WMT** | **(6.52, 4.30－8.73, p<0.001*)** | **9** | AC vs. Theta | (0.30, -0.12－0.71, p=0.17) |
| **2** | **AC vs. Sham+WMT** | **(2.64, 1.19－4.09, p<0.001*)** | **10** | AC vs. SMR | (0.29, -0.18－0.75, p=0.23) |
| **3** | AC vs. Theta/Alpha | (0.94, -0.12－2.01, p=0.08) | **11** | AC vs. LB | (0.22, -0.73－1.17, p=0.66) |
| **4** | AC vs. UA+WMT | (0.88, -0.03－1.79, p=0.05) | **12** | AC vs. Sham | (0.03, -0.40－0.46, p=0.88) |
| **5** | **AC vs. Gamma** | **(0.77, 0.02－1.53, p=0.04*)** | **13** | AC vs. SC | (0.02, -0.41－0.44, p=0.94) |
| **6** | **AC vs. Alpha** | **(0.43, 0.03－0.83, p=0.03*)** | **14** | AC vs. UA+PC | (-0.02, -0.92－0.89, p=0.97) |
| **7** | AC vs. UA | (0.34, -0.10－0.78, p=0.13) | **15** | AC vs. Alpha+Theta | (-0.04, -1.20－1.11, p=0.94) |
| **8** | AC vs. PC | (0.34, -0.30－0.97, p=0.30) |  |  |  |
| **Abbreviations:** *AC,* active control; *CI,* confidence interval; *ES,* effect size; *LA,* lower alpha; *LB,* lower beta; *PC,* passive control; *SC,* silent cotnrol; *SMR,* sensorimotor rhythm; *UA,* upper alpha; *WMT,* working memory training. | | | | | |

Table S3 displays the surface under the cumulative ranking curve (SUCRA) value on WM, with the smallest-sample study removed from a dataset (Khodakarami et al., 2020). The results of SUCRA values showed that alpha+WMT (100%) was the most effective intervention to increase WM. Additionally, the SUCRA results showed the rank of control groups based on the effectiveness of interventions in evoking EEG-NFT on WM, from the largest to the smallest (PC=47.5%, sham=20.5%, SC=19.0%, and AC=18.7%).

**Table S3 The SUCRA values on WM**

| **Rank** | **Treatment** | **SUCRA value (%)** | **Rank** | **Treatment** | **SUCRA value (%)** |
| --- | --- | --- | --- | --- | --- |
| **1** | Alpha+WMT | 100 | **9** | Theta | 45.2 |
| **2** | Sham+WMT | 92.9 | **10** | SMR | 43.5 |
| **3** | UA+WMT | 74.5 | **11** | LB | 38.3 |
| **4** | Theta/Alpha | 73.8 | **12** | Alpha+Theta | 25 |
| **5** | Gamma | 71.7 | **13** | UA+PC | 23.7 |
| **6** | Alpha | 56.8 | **14** | Sham | 20.5 |
| **7** | UA | 49.1 | **15** | SC | 19 |
| **8** | PC | 47.5 | **16** | AC | 18.7 |
| **Abbreviations:** *AC,* active control; *LA,* lower alpha; *LB,* lower beta; *PC,* passive control; *SC,* silent cotnrol; *SMR,* sensorimotor rhythm; *SUCRA,* surface under the cumulative ranking curve; *UA,* upper alpha; *WMT,* working memory training. | | | | | |

Fig. S2 shows the comparison-adjusted funnel plots and Egger’s tests on WM, with the smallest-sample study removed from a dataset (Khodakarami et al., 2020). The results exhibited a relatively symmetrical distribution, indicating no publication bias in EEG-NFT on WM (p=0.24).


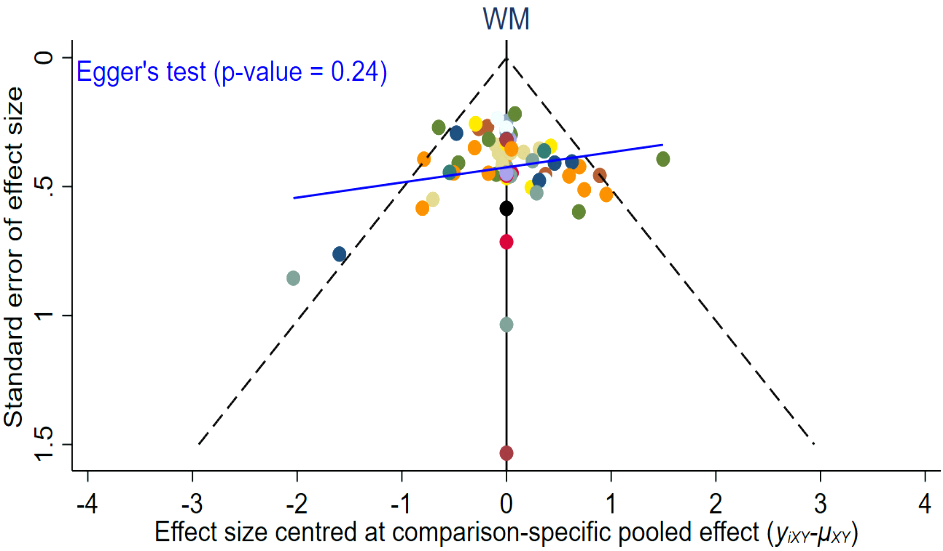


**Fig. S2 Publication bias and egger’s test for the EEG-NFT on WM.** The black line represents the null hypothesis that the study-specific effect sizes do not differ from the respective comparison-specific pooled effect estimates. The blue line represents the regression line on WM.

Fig. S3 shows NMA for EEG-based NFT on EM, with the smallest-sample study removed from a dataset (Berner et al., 2006). Twenty-three trials (688 participants) with a reference AC compared to 12 interventions (alpha, beta/theta, beta, gamma, LA/UA, PC, SMR, sham, SC, theta/LB, theta, and UA) examined the impact of the interventions.

**Episodic memory (EM)**


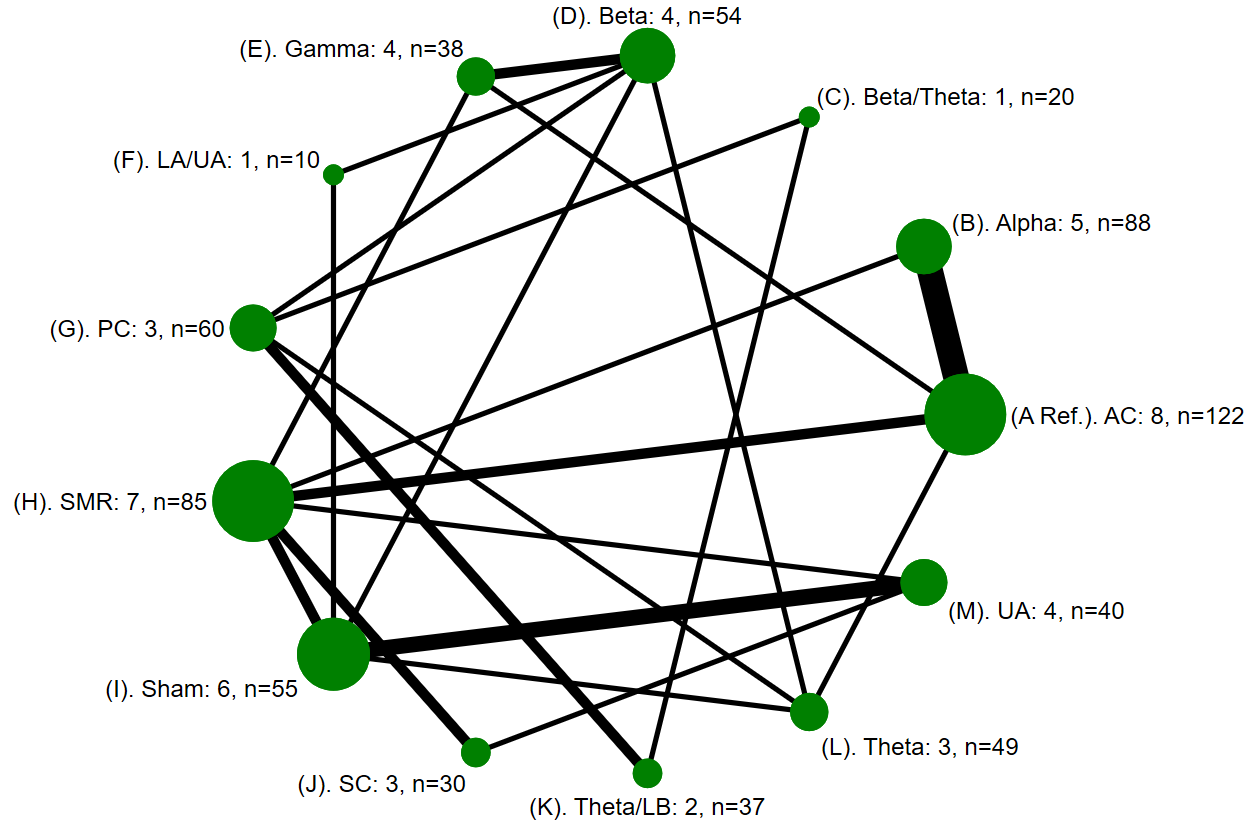


**Fig. S3 Network geometry for EEG-NFT on EM in healthy participants.** Each node indicates a particular intervention and is weighted according to the number of studies. Each edge (line connecting the nodes) is weighted according to the number of participants and directly compares the interventions it connects. Each English number respectively shows a series of information (an intervention: number of studies, n=number of participants). *AC,* active control; *LA,* lower alpha; *LB,* low beta; *PC,* passive control; *Ref.,* reference; *SC,* silent control; *SMR,* sensorimotor rhythm; *UA,* upper alpha.

Table S4 shows the inconsistency tests using loop-inconsistency models to analyse direct and indirect evidence on EM, with the smallest-sample study removed from a dataset (Berner et al., 2006). Global inconsistence showed the analysed results (χ^2^(10)=11.65, p=0.31). Side-splitting models showed great inconsistency between the beta-LA/UA (p<0.001), beta-PC (p=0.08), beta-sham (p<0.001), beta-theta (p=0.08), LA/UA-sham (p<0.001), and PC-theta (p=0.08).

**Table S4 The inconsistency tests using loop-inconsistency models on EM**

| **Side** | **Direct** | | **Indirect** | | **Difference** | | **p>z** |  | **Side** | **Direct** | | **Indirect** | | **Difference** | | **p>z** |
| --- | --- | --- | --- | --- | --- | --- | --- | --- | --- | --- | --- | --- | --- | --- | --- | --- |
|  | **Coeff.** | **SE** | **Coeff.** | **SE** | **Coeff.** | **SE** |  |  |  | **Coeff.** | **SE** | **Coeff.** | **SE** | **Coeff.** | **SE** |  |
| A B | 0.90 | 0.34 | -0.73 | 1.68 | 1.63 | 1.72 | 0.34 |  | **D L** | **1.17** | **0.67** | **-0.63** | **0.77** | **1.80** | **1.02** | **0.08** |
| A E | 0.52 | 0.82 | -0.13 | 0.72 | 0.66 | 1.09 | 0.55 |  | E H | 0.99 | 0.80 | -0.57 | 0.67 | 1.55 | 1.04 | 0.14 |
| A H | 0.36 | 0.55 | 0.00 | 0.72 | 0.37 | 0.91 | 0.69 |  | **F I** | **-1.44** | **0.68** | **4.41** | **1.68** | **-5.85** | **1.86** | **0.00** |
| A L | 0.32 | 0.75 | 1.19 | 0.81 | -0.87 | 1.10 | 0.43 |  | G K | 0.04 | 0.52 | 2.28 | 449.19 | -2.25 | 449.19 | 1.00 |
| B H | -0.49 | 0.75 | -0.72 | 0.70 | 0.23 | 1.02 | 0.82 |  | **G L** | **2.30** | **0.69** | **-1.30** | **1.92** | **3.61** | **2.05** | **0.08** |
| C G | 0.00 | 0.75 | -1.19 | 2.02 | 1.19 | 2.15 | 0.58 |  | H I | 0.16 | 0.55 | -1.01 | 0.67 | 1.17 | 0.87 | 0.18 |
| C K | -0.25 | 0.75 | 0.94 | 2.02 | -1.19 | 2.15 | 0.58 |  | H J | -0.32 | 0.58 | -0.88 | 1.01 | 0.55 | 1.16 | 0.63 |
| D E | 0.08 | 0.58 | -0.85 | 0.93 | 0.93 | 1.10 | 0.40 |  | H M | -0.21 | 0.82 | -0.07 | 0.65 | -0.14 | 1.05 | 0.89 |
| **D F** | **-0.66** | **0.66** | **5.19** | **1.71** | **-5.85** | **1.86** | **0.00** |  | I L | 0.24 | 0.86 | 1.23 | 0.75 | -0.99 | 1.14 | 0.38 |
| **D G** | **-1.13** | **0.67** | **-4.73** | **1.95** | **3.60** | **2.05** | **0.08** |  | I M | 0.05 | 0.47 | 1.00 | 1.11 | -0.95 | 1.20 | 0.43 |
| **D I** | **-2.10** | **0.72** | **0.83** | **0.59** | **-2.93** | **0.93** | **0.00** |  | J M | 0.62 | 0.82 | 0.06 | 0.82 | 0.55 | 1.16 | 0.63 |
| **Notes:** A=AC, B=alpha, C=beta/theta, D=beta, E=gamma, F=LA/UA, G=PC, H=SMR, I=sham, J=SC, K=theta/LB, L=theta, and M=UA.  **Abbreviations:** *Coeff.,* coefficient; *CI,* confidence interval; *SE,* standard error. | | | | | | | | | | | | | | | | |

Table S5 displays the consistency assessment showing a pairwise comparison of AC versus included interventions in terms of EEG-NFT on EM, with the smallest-sample study removed from a dataset (Berner et al., 2006). The results showed that a reference (AC) compared to alpha was significant difference. Specially, AC versus NFT of alpha activity exhibited no any inconsistency between the direct and indirect effect from one another in Table S4, indicating that the efficacy of alpha NFT was accepted in the consistency assessment.

**Table S5 The consistency assessment on EM**

| **Rank of ES** | **Interventions** | **(ES, 95% CI, p-value)** | **Rank of ES** | **Interventions** | **(ES, 95% CI, p-value)** |
| --- | --- | --- | --- | --- | --- |
| **1** | **AC vs. Alpha** | **(0.84, 0.18－1.49, p=0.01*)** | **7** | AC vs. UA | (0.11, -1.09－1.31, p=0.86) |
| **2** | AC vs. Theta | (0.72, -0.35－1.79, p=0.19) | **8** | AC vs. Sham | (-0.09, -1.15－0.97, p=0.87) |
| **3** | AC vs. LA/UA | (0.47, -1.19－2.13, p=0.58) | **9** | AC vs. SC | (-0.23, -1.48－1.02, p=0.72) |
| **4** | AC vs. Beta | (0.33, -0.80－1.47, p=0.57) | **10** | AC vs. Beta/Theta | (-1.02, -3.09－1.05, p=0.33) |
| **5** | AC vs. SMR | (0.23, -0.61－1.06, p=0.59) | **11** | AC vs. Theta/LB | (-1.13, -3.01－0.75, p=0.24) |
| **6** | AC vs. Gamma | (0.16, -0.89－1.197, p=0.77) | **12** | AC vs. PC | (-1.17, -2.75－0.41, p=0.15) |
| **Abbreviations:** *AC,* active control; *CI,* confidence interval; *ES,* effect size; *LA,* lower alpha; *LB,* lower beta; *PC,* passive control; *SC,* silent cotnrol; *SMR,* sensorimotor rhythm; *UA,* upper alpha. | | | | | |

Table S6 displays the SUCRA value on EM, with the smallest-sample study removed from a dataset (Berner et al., 2006). The results of SUCRA values showed that alpha (86.9%) was the most effective intervention to increase EM. Additionally, the SUCRA results showed the rank of control groups based on the effectiveness of interventions in evoking EEG-NFT on EM, from the largest to the smallest (AC=46.8%, sham=42.6%, SC=37.2%, and PC=12.3%).

**Table S6 The SUCRA values on EM**

| **Rank** | **Treatment** | **SUCRA value (%)** | **Rank** | **Treatment** | **SUCRA value (%)** |
| --- | --- | --- | --- | --- | --- |
| **1** | Alpha | 86.9 | **8** | AC | 46.8 |
| **2** | Theta | 83.2 | **9** | Sham | 42.6 |
| **3** | LA/UA | 69 | **10** | SC | 37.2 |
| **4** | Beta | 65.6 | **11** | Beta/Theta | 20.1 |
| **5** | SMR | 60.8 | **12** | Theta/LB | 15.2 |
| **6** | Gamma | 55.9 | **13** | PC | 12.3 |
| **7** | UA | 54.2 |  |  |  |
| **Abbreviations:** *AC,* active control; *LA,* lower alpha; *LB,* lower beta; *PC,* passive control; *SC,* silent cotnrol; *SMR,* sensorimotor rhythm; *SUCRA,* surface under the cumulative ranking curve; *UA,* upper alpha. | | | | | |

Fig. S4 shows the comparison-adjusted funnel plots and Egger’s tests on EM, with the smallest-sample study removed from a dataset (Berner et al., 2006). The results exhibited a relatively symmetrical distribution, indicating no publication bias in EEG-NFT on EM (p=0.19).


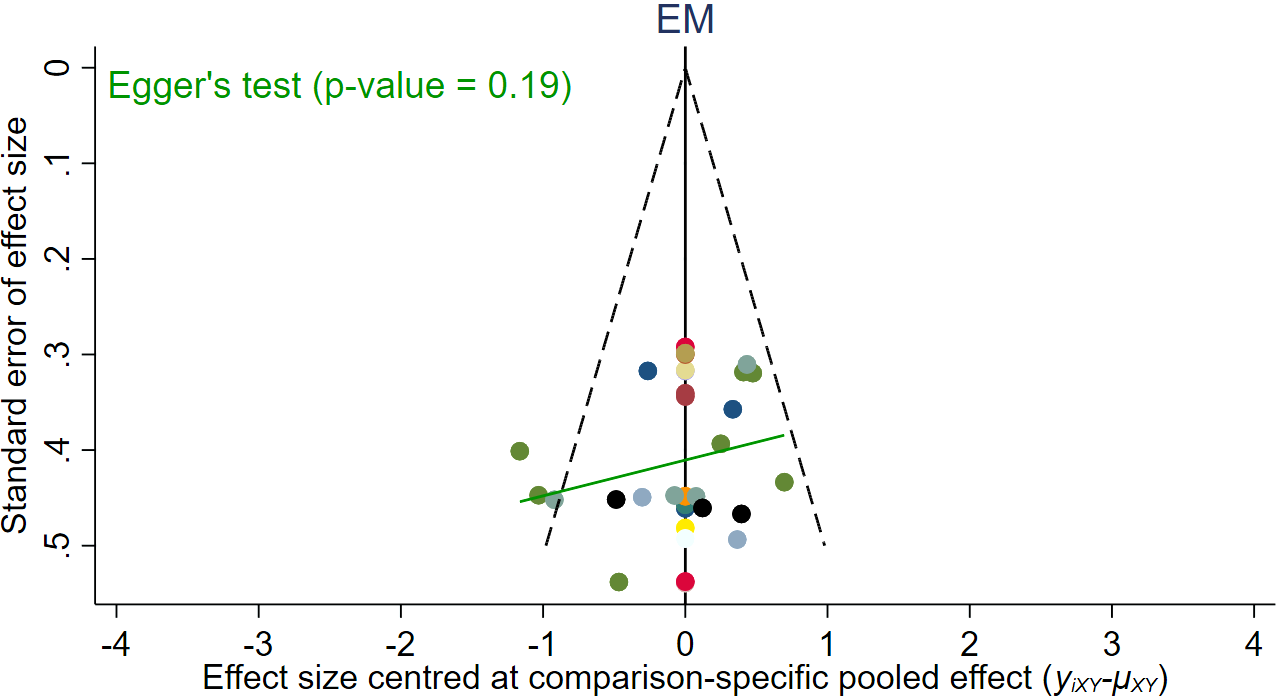


**Fig. S4 Publication bias and egger’s test for the EEG-NFT on EM.** The black line represents the null hypothesis that the study-specific effect sizes do not differ from the respective comparison-specific pooled effect estimates. The green line represents the regression line on EM.
